# Prevalence, awareness, treatment and control of hypertension among ethnoracial minorities in France: results from the CONSTANCES cohort

**DOI:** 10.1136/bmjopen-2024-097800

**Published:** 2025-09-22

**Authors:** Léna Silberzan, Emmanuel Wiernik, Nathalie Bajos, Michelle Kelly-Irving

**Affiliations:** 1 CERPOP-UMR 1295, Université de Toulouse, INSERM, Toulouse, France; 2 UMR 8156, INSERM, Paris, France; 3 UMS 011 « Population-based Cohorts Unit », INSERM, Paris, France; 4 UMR 1027, INSERM, Paris, France

**Keywords:** Awareness, Blood Pressure, Hypertension, Chronic Disease, Prevalence

## Abstract

**Abstract:**

**Objectives:**

Race/ethnicity, combined with sex, is an important determinant of hypertension prevalence and management in high-income countries, but data for France are lacking. This study aims to explore hypertension prevalence and each stage of the cascade of care (*i.e.*, awareness, treatment, and control), at the intersection of sex and race/ethnicity in a French cohort.

**Design:**

We used data from the population-based CONSTANCES cohort, linked with the French National Health Data System.

**Participants:**

180 459 individuals were included, aged 18–69 (mean age 47, SD: 13), among which 53% (n=95 395) women and 81% (n=145 983) of the majority group, and 4.9% (n=8 775) of North African, 1.2% (n=2 220) of sub-Saharan African (SSA), 1.2% (n=2 204) of Asian, 1.4% (n=4 462) of Overseas France *départements* and regions (DROMs) and 10% of European and other descents. Among these 180 459 individuals, 54 009 (29.9%) had hypertension.

**Primary and secondary outcome measures:**

Migration status was used as a proxy for race/ethnicity. Age-standardized hypertension rates were estimated by sex and race/ethnicity. Multinomial logistic regressions, adjusted for age, were used to compare ethnoracial differences in the cascade of care.

**Results:**

Individuals from SSA or DROMs had higher prevalence rates than the majority group, especially among women (37.6% and 26.8% *vs* 20.8%, respectively). These groups also had higher odds of entering a hypertension care path, although women from SSA tended to remain treated, instead of achieving control (OR 1.39 (0.99 to 1.96)). Women of Europe and others (OR 1.46 (1.14 to 1.87)) and men originating from Asia (OR 1.85 (1.03 to 3.33)) were more likely to remain at the awareness step.

**Conclusion:**

Race/ethnicity impacts hypertension prevalence and management in France, with variations by sex. Our findings underscore the necessity to consider these results when designing intervention strategies to reduce the burden of uncontrolled hypertension.

**STRENGTHS AND LIMITATIONS OF THIS STUDY**

- Based on a large sample in the general population, with clinical measures, and linked to the French National Health Data System, providing exhaustive tracing of all outpatient data (*e.g.*, prescription reimbursements).
- Quantifies the intersection of sex and race/ethnicity, adjusted for age, on all the steps of the cascade of care.
- Uses multinomial analysis that makes it possible to work on a common denominator (*i.e.,* individuals with hypertension) and compare ORs at each step of the cascade.
- Detailed ethnoracial sub-categories (sub-Saharan Africans, Overseas France *départements* and regions, North Africans, Asians, Europeans and others, majority group).
- Uses a proxy for race/ethnicity, instead of self-reported race/ethnicity.

## Introduction

 High blood pressure (BP) is a leading risk factor for death and disability,^1^ across all ages and ethnoracial groups.^2 3^ Although hypertension can easily be detected in community and care facilities and effective drugs are available at fairly low costs,^4^ control rates remain low, including in high-income countries.^4^ Hypertension management, sequentially divided into awareness, treatment, and control, and known as the hypertension cascade of care (or care cascade), is therefore instrumental to unveiling the origins of uncontrolled hypertension and to understanding existing health inequalities.

Studies in high-income countries,^5^ including France,^6^ reveal sex and age differences in hypertension prevalence and across the cascade of care. Women have lower prevalence rates, but higher awareness, treatment and control rates, whereas older individuals tend to have higher prevalence, awareness and treatment rates, but lower control rates on average.^5^

Differences also exist across ethnoracial groups.^7 8^ Examining the social determinants of hypertension prevalence and management at the intersection of these different categories of sex, age, and race/ethnicity may reveal important new areas for healthcare prevention and management strategies. For instance, a study in the USA finds that, after adjusting for age and sex,^7^ compared with White Americans, Black Americans have higher prevalence and lower control rates, Asian Americans have lower awareness rates, and Hispanic Americans have lower awareness and treatment rates. When a moderating effect of sex is examined, this study also finds that Black women have increased hypertension prevalence compared to Black men. However, these results are difficult to generalize to other national contexts, and there have been recent calls for similar studies in European countries. ^3^While those studies are still scarce, a systematic review,^8^ mainly using data from cohorts in the UK and the Netherlands (none from France), finds race/ethnicity inequalities among men and women, although age could not be taken into account.

In this context, the lack of research on hypertension prevalence and management across ethnoracial groups in France, the second most populated country in the European Union, where one in three adults has hypertension,^9^ and less than one in four of them reaches control,^6^ is concerning. The restrictive national legal framework on data collection contributes to a shortage of ethnoracial data in health surveys, despite calls for change.^10 11^ However, in a country where immigrants and natives from Overseas France *départements* and regions (DROMs) (Overseas France consists of French territories located outside Europe) make up around 14% (among which around 8% from DROMs, 30% from North Africa, 13% from sub-Saharan Africa (SSA), 19% from Europe and 11% from Asia) of the metropolitan French population (metropolitan France, also know as mainland France, is the part of France located in Europe, as opposed to DROMs), and descendants of immigrants and of natives from DROMs around 13% (among which around 10% from DROMs, 6% SSA, 36% from Europe and 7% from Asia),^12^ migration status is a different variable that may act as an imperfect proxy for race/ethnicity. It is strongly correlated with race/ethnicity and more commonly collected and available in routine data in France and can help identify populations that may be disproportionately exposed to discrimination related to racism and xenophobia.^13^ As such, to examine ethnoracial health inequalities in France, migration status may offer a pragmatic solution and provide insight into an otherwise invisible set of mechanisms underlying health inequalities.^13 14^

Only two clinical studies have analysed hypertension by migration status in metropolitan France, using similar cross-sectional study settings in a hypertension hospital department.^15 16^ Whatever the sex, no significant difference for BP levels and antihypertensive treatment was found between the 719 patients born in North Africa and 3 558 patients born in Europe (France included) in the 2010 study; however, systolic BP levels were higher among the 539 SSA patients, despite higher treatment rates, when compared with the 2 610 patients born in Europe (France included) in the 2007 study.^16^ However, these results cannot be generalized to the general population, the studies are limited to North Africans and SSA and do not investigate other migratory groups (such as DROMs or Asians), and being referred to a specialist hypertension unit may be socially determined and associated with greater hypertension awareness.

To address existing gaps in the evidence from France, our study describes the prevalence of hypertension and each stage of the hypertension cascade of care within the general population, at the intersection of sex, age and a wide range of ethnoracial groups.

## Methods

### CONSTANCES

The CONSTANCES cohort is a French prospective cohort composed of randomly selected adults aged 18–69 from the general population. At baseline, all participants were affiliated with the French National Health Insurance Fund database, which covers 85% of the general French population, following a stratified sampling based on age, sex, employment, occupation and region.^17^ All participants provided written consent.

At inclusion, participants visited health screening centres (HSCs) for comprehensive health assessments, including doctor-administered questionnaires and three standardized BP readings. Standardized Operating Procedures for data collection were provided by the CONSTANCES study team.^18^ Yearly inspected calibrated oscillometric automated devices (OMRON1 705CP-II or OMRON1 705IT) with an appropriately sized cuff were used. BP measurements were conducted three times after a 5-min rest in a lying position. The first measurement was taken on the right arm, the second on the left arm with a 1-min interval, and the third on the reference arm (the arm with the highest value) after another 1-min interval. The mean of the two measurements on the reference arm was used as the systolic and diastolic BP.

The study participants’ data were linked to the French National Health Data System (SNDS), providing exhaustive tracing of all outpatient data (*e.g.*, prescription reimbursements).

### Definitions

Hypertension was defined as having a BP≥140/90 mm Hg and/or having been reimbursed for at least one antihypertensive medication in the past 6 months.

Awareness was determined by reported history of hypertension in response to the doctor-administered questionnaire.

Treatment was assessed using information derived from the SNDS database. Hypertensive patients were considered treated if reimbursed for at least one box of antihypertensive medication within 6 months before inclusion.

Control was determined as an average BP <140/90 mm Hg.

**Figure 1:**
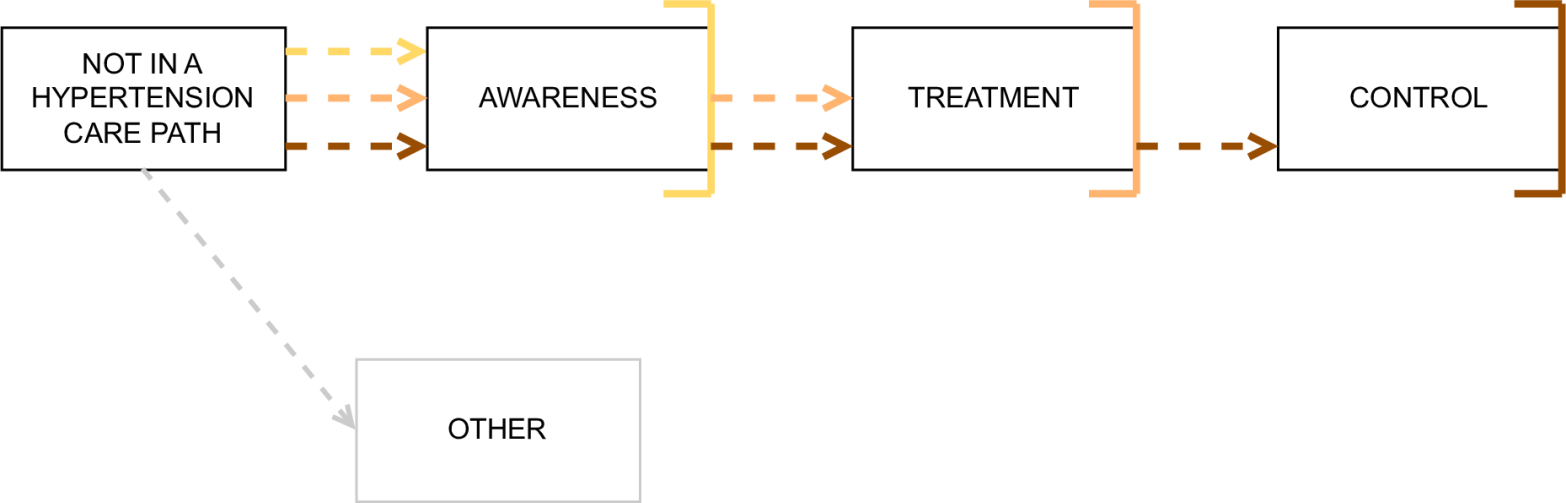
Hypertension management patterns. Notes: each individual can only be in one of the following steps of hypertension management. ‘Not in a hypertension care path’: no awareness, no treatment and no control. ‘Awareness’: awareness, but no treatment and no control (yellow path). ‘Treatment’: awareness and treatment, but no control (orange path). ‘Control’: awareness, treatment and control (brown path). ‘Other’: hypertension care trajectories that are not compatible with the cascade of care framework (no awareness, but treatment) (grey path).

In our study, individuals with hypertension are attributed to a specific step of the cascade of care framework depending on their awareness, treatment and control status (figure 1). Throughout this paper, unless stated otherwise, and except for the ‘Other’ path, treatment presupposes awareness, and control presupposes treatment. In the cascade of care framework, we determine that individuals are in the ‘Other’ path if they reach treatment (regardless of their control status), without reaching awareness. Furthermore, unless stated otherwise, a person at the ‘awareness’ step is considered not to have achieved treatment or control in our study (similarly, a person at the ‘treatment’ step is considered not to have achieved control).

### Study participants

We pooled data from 205 203 individuals included in the CONSTANCES cohort from February 2012 to May 2021. We excluded individuals who did not visit a HSC (n=8 899), individuals whose data were not available through linkage with the SNDS database, did not have three valid measures of hypertension or had missing data on hypertension awareness (n=12 780), as well as participants with missing data on migration status (n=2 173). Pregnant women were not included (n=892) to avoid specific cases of congenital hypertension. The final sample consisted of 180 459 individuals (aged 18–69), of whom 29.9% (n=54 009) had hypertension (online supplemental figure 1).

### Patient and public involvement

Patients or the public were not involved in the design, or conduct, or reporting, or dissemination plans of our research.

### Measures and outcomes

Age and sex were collected in administrative records.

Migration status was used as a proxy for race/ethnicity and was assessed using the participant’s response to questions about their parents’ and their own geographical area of origin (eight-item question: metropolitan France/DROM/Europe/North Africa/SSA/Asia/Other/cannot answer) and nationality (online supplemental figure 2). Doing so, the ‘majority group’ was defined as all individuals residing in metropolitan France who are neither immigrants nor DROM natives (DROMs consist of French territories located outside Europe, largely made up of the remaining parts of the former French colonial empire that continued to be integrated into the French state under different legal statuses after decolonisation). Among these territories, the most populated ones (over 200 000 inhabitants in 2021) are Guadeloupe, Martinique, French Guiana, Réunion, Mayotte, French Polynesia and New Caledonia), nor descendants of these populations. The term ‘majority group’ captures both a statistical overrepresentation and an advantaged position within the stratified structure of French society^19^ (in contrast, group sizes are smaller in the other migratory groups, which can be referred to as ‘minorities’, and individuals in these groups are treated differently in France with respect to their origin^19^). In France, the experience of discrimination and racism more directly concerns immigrants and descendants from North Africa, SSA, Asia, along with DROM natives and descendants.^19^ For this reason, along with statistical reasons, immigrants and descendants of immigrants from the same geographical area of origin were grouped together, as well as DROM natives and descendants^19^ (European and the ‘other’ geographical origin were also grouped together, as it is likely that they experience less direct discriminations linked to racism and xenophobia). Migration status, used as a proxy for race/ethnicity, was therefore coded as follows: majority group, SSA group, DROM group, North African group, Europe and other group and Asian group.

Education level was divided into three categories corresponding to educational attainment within the French education system: up to high school diploma (12 years), undergraduate education (14–15 years), and postgraduate education (16 years and more).

Overweight/obesity was determined using the HSC weight and height measurements.^18^ Weight was measured using a non-automatic weighing instrument, adhering to the International Organization of Legal Metrology recommendations (OIML R 76–1, Edition 2006). Standing height was measured using a fixed stadiometer with a vertical backboard and a moveable headboard to the nearest 0.1 cm. Body mass index (BMI) (weight (kg)/height (m²)) was computed and the WHO criterion was used to identify respondents with overweight (25≤BMI<30 kg/m²) and obesity (BMI≥30 kg/m²).

Dietary assessment was done through a self-answered validated 52-item food frequency questionnaire. In order to have a robust score, we used six of the eight DASH (Dietary Approaches to Stop Hypertension) food or nutrients categories available in our dataset for which consumption should be increased (vegetables, fruits, nuts and legumes, low-fat dairy) or reduced (sodium, red and processed meats). Consumption of each component was divided into quintiles and assigned one to five points according to a sex-specific intake ranking.^20^ The adapted DASH score was divided into terciles for the analysis.

To determine a 5-year preceding inclusion average number of visits to a general practitioner (GP), we used pooled data from 2007 to 2021 from the SNDS database. For each participant, we summed all reimbursements for visits to a GP in the 5-year preceding inclusion and divided this number by five to obtain an average yearly visit ratio.

Individuals with type II diabetes were individualized when they reported type II diabetes, received anti-diabetic medication in the SNDS database or had a fasting blood glucose concentration >7 mmol/L.

Dyslipidaemia was assessed at inclusion with a fasting blood sample. Total cholesterol and triglycerides were all measured within hours of sampling. An individual was considered to have dyslipidaemia when they reported having treated hypercholesterolaemia or treated hypertriglyceridaemia, or when they received at least 1 box of a lipid-lowering treatment during the 6 months preceding the clinical examination or if they had fasting plasma total-cholesterol or triglycerides level of ≥6.61 mmol/L (255 mg/dL) or >1.7 mmol/L (150 mg/dL), respectively.^20^

History of other cardiovascular diseases (CVDs) (namely coronary disease, myocardial infarction, stroke, transient ischaemic attack, peripheral artery disease, heart failure) was either reported when answering the doctor-administered questionnaire or identified in the SNDS. Using self-reported data in CONSTANCES enabled us to complete the registry data (SNDS) that only recorded medical reimbursements or hospitalisations for one of the aforementioned CVD between 2007 and 2021.

Self-reported smoking status was classified into two categories: current smoker and non-smoker (including former smoker).

Multiple imputation techniques were applied to account for missing data (<5%) on education level, obesity/overweight status, DASH score and tobacco use.

### Statistical analyses

Age-standardized hypertension rates by sex and race/ethnicity were estimated with the direct age-standardisation method, using the 2016 French census distribution (grouped into 18–34, 35–44, 45–54, 55–64 and 65–69 years).

We then described the distribution across the cascade of care steps for each ethnoracial group, stratified by sex.

We used multinomial logistic regressions, adjusted for age, to compare ethnoracial differences in the cascade of care distribution. To examine the association between race/ethnicity and the hypertension cascade of care among men and women, we conducted separate analyses by sex.

As the objective of our study was to provide a descriptive overview of the distribution prevalence of hypertension and each stage of the hypertension cascade of care at the intersection of sex, age and race/ethnicity, we did not investigate causal mechanisms. Therefore, we did not adjust for other variables than age and race/ethnicity, to avoid overadjustment bias.^21^

Furthermore, while ORs are a valid measure of association in logistic regression, they become increasingly difficult to interpret as intuitive measures of risk when the outcome is common (as can be the case in our analyses). In such cases, ORs can substantially overstate the strength of association compared with differences in predicted probabilities, potentially leading to misleading conclusions if interpreted as direct measures of risk. That is why we added marginal predicted probabilities (adjusted for age) to provide a more intuitive and interpretable summary of ethnoracial differences (by sex) in the hypertension cascade of care.

In our study, given the relatively small sample sizes in some categories, we discussed results at both 0.95, but also 0.90 confidence levels to explore potential trends.

## Results

Baseline characteristics of the CONSTANCES cohort (n=180 459) by race/ethnicity and sex are shown in online supplemental table 1. Briefly, women made up 53% of the cohort, and 19% of participants belonged to an ethnoracial minority: 4.9% (n=8 775) of North African, 1.2% (n=2 220) of SSA, 1.2% (n=2 204) of Asian, 1.4% (n=4 462) of DROMs, 10% of European and other (n=18 815) descents, with no significant differences between women and men. The mean age was 47 years old (SD: 13). Women from SSA and Asia were the youngest groups, with mean ages of 40 years (SD: 12) and 41 years (SD: 12), respectively. Men from the majority group were the oldest, with a mean age of 48 years (SD: 13). More than half of the participants in the following groups—men and women from SSA, men from the majority group, and men and women from DROMs, North Africa, and Europe and Other regions—were either overweight or obese. Descriptive data on the hypertensive population, as well as the subpopulations of those aware (whatever their treatment or control status) and aware and treated (whatever their control status), can be found in online supplemental tables 2–4, respectively.

### Prevalence

**Figure 2:**
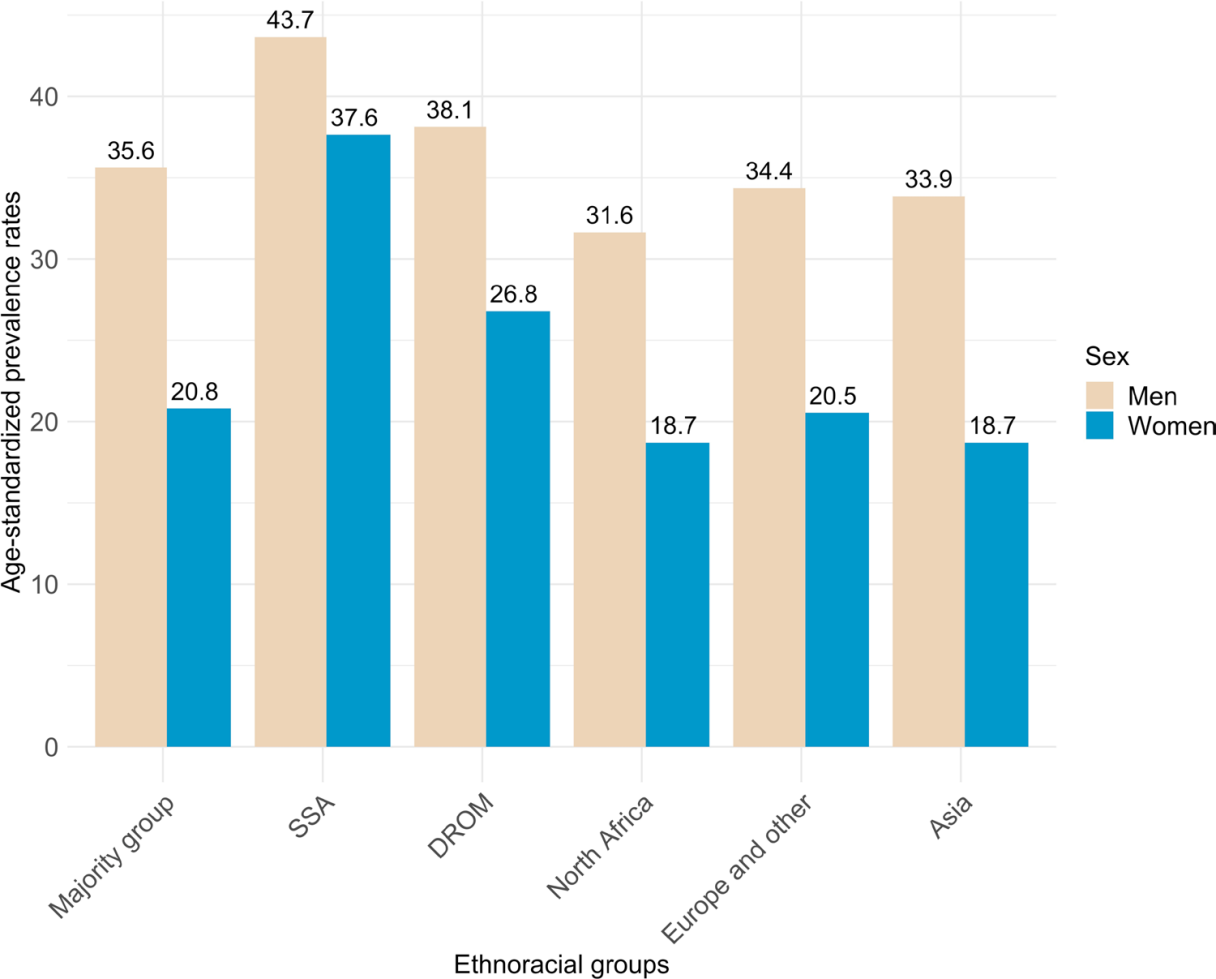
Age-standardized hypertension prevalence rates by sex and race/ethnicity. Notes: SSA, sub-Saharan African group; DROM, Overseas France *départements* and regions group.

Among the 180 459 individuals included in our study, 29.9% (n=54 009) had hypertension (mean age 55 (SD:11), 39% women and 3.6% (n=1 933) of North African, 1.4% (n=758) of SSA, 0.8% (n=429) of Asian, 1.3% (n=697) of DROMs, 10% of European and other (n=5 564) descents, and 83% (n=44 628) of the majority group). Age-standardized hypertension rates were higher among men (35.4% of men *vs* 20.9% of women); and within the SSA (40.3%) and DROM (31.6%) groups, compared with the majority group (27.8%) (online supplemental table 5). SSA and DROM women had higher prevalence rates than women from the majority group (37.6% and 26.8% *vs* 20.8%, respectively), with narrower sex gaps in these groups (5.8% and 11.3%, respectively, *vs* 14.6% for the majority group) (figure 2). The differential effect of sex on race/ethnicity for these two groups is confirmed when adding a multiplicative interaction term in a logistic regression (online supplemental table 6).

### Cascade of care

**Figure 3:**
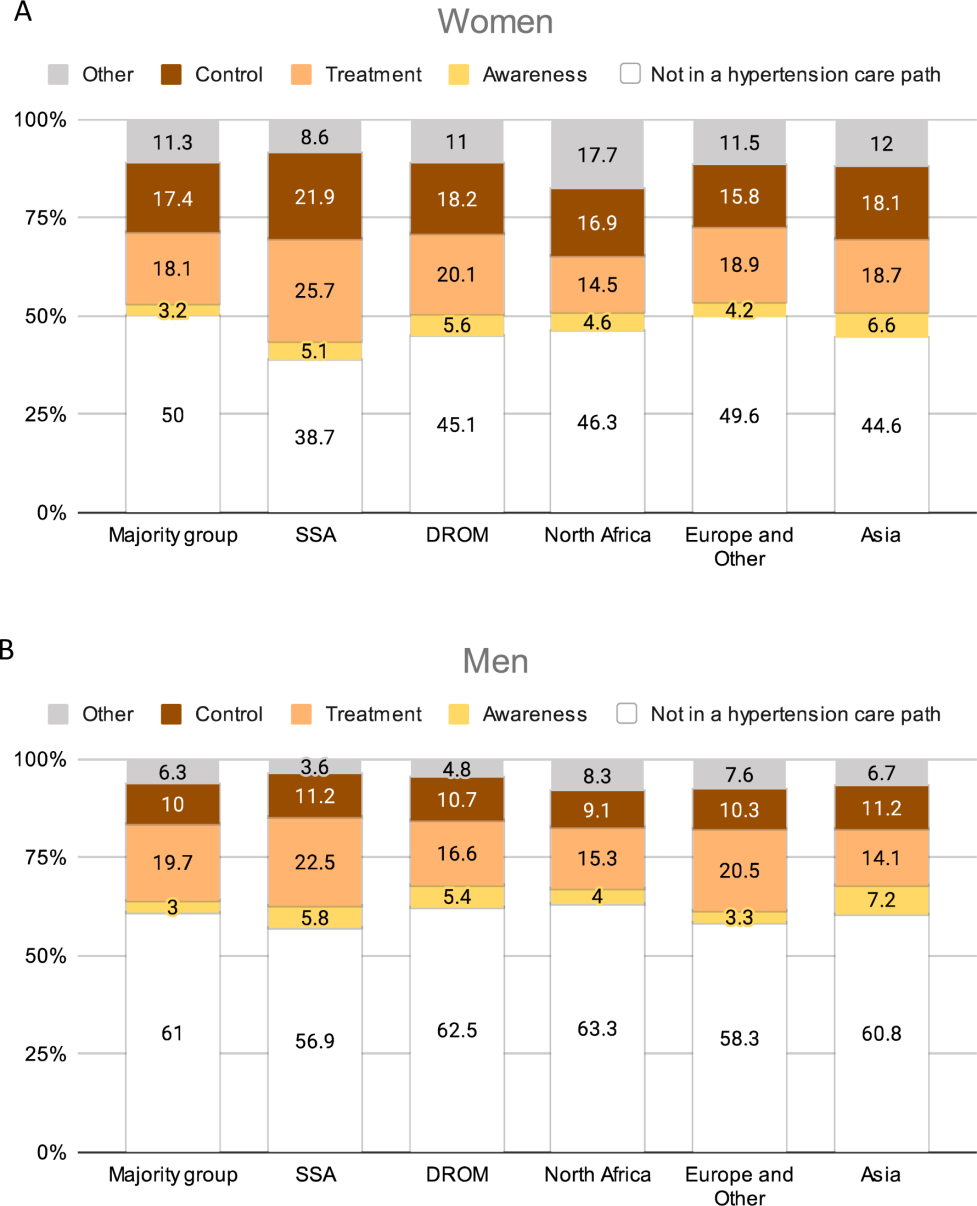
Hypertension management by race/ethnicity, among women (**A**) and men (**B**). Each individual can only be in one of the following steps of hypertension management. ‘Not in a hypertension care path’: no awareness, no treatment and no control. ‘Awareness’: awareness, but no treatment and no control. ‘Treatment’: awareness and treatment, but no control. ‘Control’: awareness, treatment and control. ‘Other’: hypertension care trajectories that are not compatible with the cascade of care framework (no awareness, but treatment). SSA, sub-Saharan African group; DROM, Overseas France *départements* and regions group.

Among hypertensive individuals, 56.4% were not in a hypertension care path, 3.3% remained aware, 19.0% remained aware and treated and 12.9% achieved control. 8.4% were on care trajectories that were not compatible with the cascade of care framework, considered as ‘Other’, with differences across sexes and race/ethnicity (figure 3).

**Figure 4:**
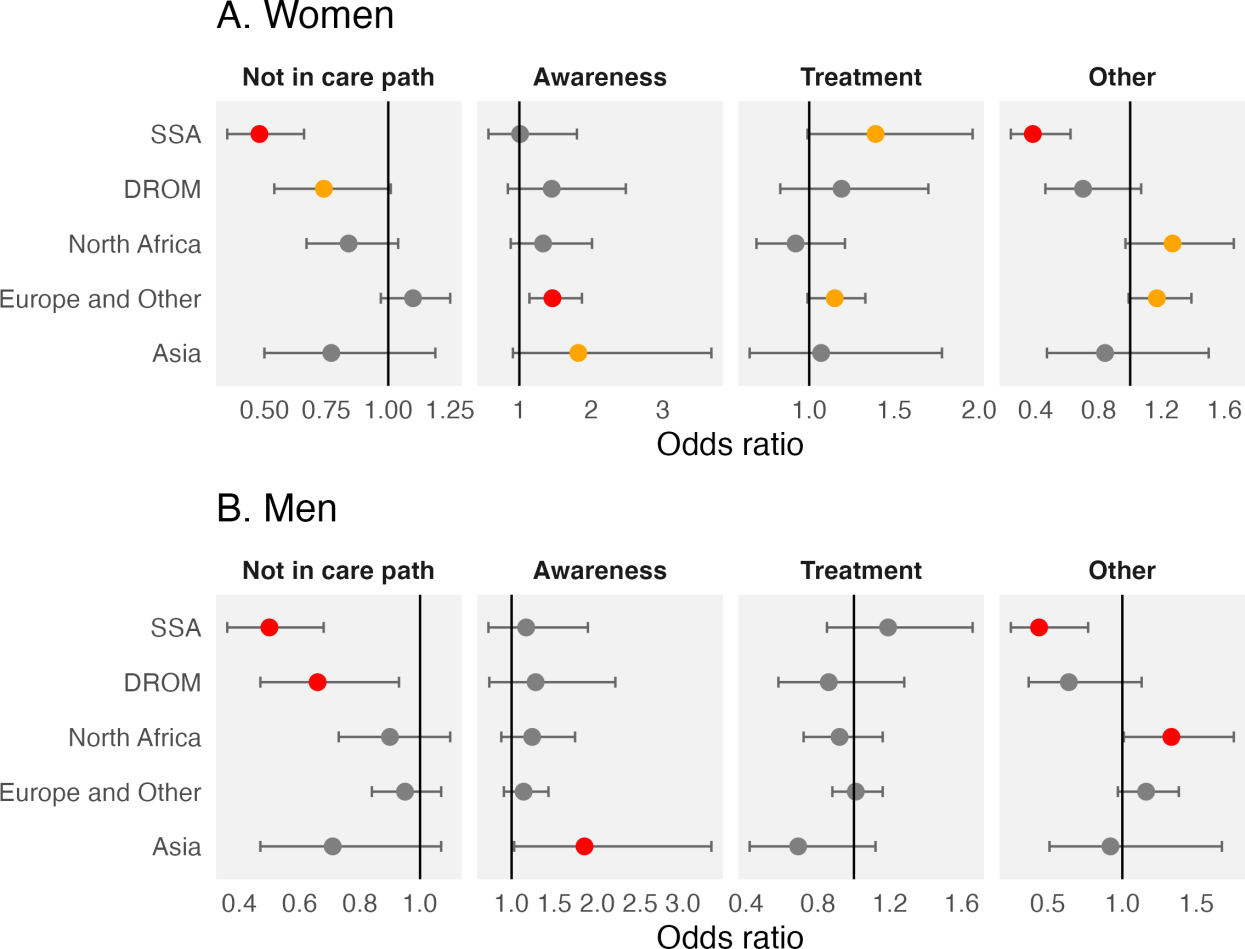
Odds ratios of being in different steps of the hypertension cascade of care, compared with being controlled among women (**A**) and men (**B**), according to ethnicity, adjusted for age. For both sexes, the ethnoracial reference group is ‘majority group’. The reference hypertension management step is ‘control’ (ie, awareness, treatment and control). In grey, p≥0.1; in orange, 0.05≤p<0.1; in red, p<0.05. Notes: each individual can only be in one of the following steps of hypertension management. ‘Not in a hypertension care path’: no awareness, no treatment and no control. ‘Awareness’: awareness, but no treatment and no control. ‘Treatment’: awareness and treatment, but no control. ‘Control’: awareness, treatment and control. ‘Other’: hypertension care trajectories that are not compatible with the cascade of care framework (no awareness, but treatment). SSA, sub-Saharan African group; DROM, Overseas France *départements* and regions group.

The age-adjusted odds of being at any one step of the cascade of care in terms of ethnoracial group, based on multinomial regression analyses, are presented in figure 4 and in online supplemental table 7a,b. All comparisons take the control step as the reference in the following results. We present ORs, completed by marginal predicted probabilities (adjusted for age) that can be found in online supplemental table 8a,b.

North African men had higher odds than those of the majority group to be in the ‘other’ management path (*i.e.*, no awareness, but treatment) (age-adjusted marginal predicted probabilities: 9.2% *vs* 6.5%, OR=1.33 (95% CI: 1.01 to 1.75), p=0.040). A similar pattern was observed for North African women (14.7% *vs* 10.9%, 1.27 (0.97 to 1.66), p=0.081), though this did not reach conventional levels of statistical significance (α=0.05). For North African men and women, the odds of remaining in the awareness or the treatment step, or not being in a hypertension care path, were not statistically significantly different from the majority group.

When compared with their counterparts of the majority group, SSA women and men had lower odds of not being in a hypertension care path (women: 34.1% *vs* 51.8%, 0.48 (0.35 to 0.66), p<0.001; men: 47.4% *vs* 64.4%, 0.50 (0.36 to 0.68), p<0.001) or in the ‘other’ management path (women: 5.7% *vs* 10.9%, 0.38 (0.24 to 0.62), p<0.001; men: 4.3% *vs* 6.5%, 0.44 (0.25 to 0.77), p=0.004). In other words, compared with the majority group, the SSA group had a higher odds of being included in a ‘typical’ hypertension care path (*i.e.*, one compatible with the cascade of care framework) and a lower odds of being in the ‘other’ path (*i.e.*, an alternative care path), compared with being controlled. Furthermore, SSA women also tended to remain treated but not controlled (31.6% *vs* 16.6%, 1.39 (0.99 to 1.96), p=0.059). This was not the case for SSA men.

To a lesser extent, compared with their counterparts of the majority group, people from DROMs had a lower odds of not being included in a hypertension care path (*i.e.*, a higher odds of being included in a hypertension care path) (women: 43.6% *vs* 51.8%, 0.74 (0.54 to 1.01), p=0.055; men: 57.2% *vs* 64.4%, 0.66 (0.47 to 0.93), p=0.019). Women of this group were also less likely to be in the ‘other’ group (8.7% *vs* 10.9%, 0.70 (0.46 to 1.07), p=0.10)

Asian men had a higher odds of remaining aware (7.6% *vs* 3.2%, 1.85 (1.03 to 3.33), p=0.041), as well as women of European and other groups (4.3% *vs* 3.3%, 1.46 (1.14 to 1.87), p=0.002). Women of this group also had higher odds of remaining treated (17.1% *vs* 16.6%, 1.15 (0.99 to 1.33), p=0.075).

## Discussion

Our study shows the diversity in hypertension prevalence and management patterns by sex and race/ethnicity, while accounting for age, in a large population-based study in France. We find that 29.9% (n=54 009) of respondents have hypertension, aligning with studies representative of the French adult population.^6 9^ People originating from SSA or DROMs have higher prevalence rates compared with the majority group, especially among women. These groups also have higher odds than the majority group to enter a hypertension care path. In our study, most respondents with hypertension are not aware, hence not treated nor controlled (54.6%), and only 12.9% are aware, treated and eventually controlled. We also observe that 8.4% do not fall in the traditional health cascade of care categories.

Compared with the other sex-stratified ethnoracial groups, men and women from the SSA group (DROM group, to a lesser extent) have higher prevalence rates. While ethnoracial disparities in hypertension prevalence are documented in other high-income countries, such as the USA,^7^ the Netherlands or the UK,^22 23^ it is not the case for France. These studies show that individuals of African origin in Europe (African Americans in the US, respectively) consistently have higher hypertension rates. While there may be genetic markers of predisposition to hypertension in African populations, although intensive investigations into genetic links have yielded less conclusive results than anticipated^2^, they are not sufficient on their own to cause hypertension or elevate risk.^24^ Social factors play a critical role in this higher burden.^22 24^ Specifically, structural racism,^25^ socioeconomic disadvantage, discrimination, differential treatments in the healthcare system and chronic stress^26^ increase hypertension risk—such as overweight and obesity—^27^ and underlie the increased risk of high BP from younger ages in these populations. Not only can the latter factors lead to higher BP levels, but according to the concept of weathering,^28^ their cumulative impact leads to premature ageing and deterioration of health, leading to higher BP rates earlier in life among these marginalized populations.^29^ A French study in 2020 in the general population shows that both first- and second-generation SSA immigrants and DROM natives report the highest discrimination rates, at least twice as much as the majority group,^30^ which could contribute to explaining our results. Furthermore, we found that SSA women had particularly high hypertension rates, which is in accordance with studies in Europe,^22^ Canada^31^ and the USA.^26^ The latter study, using the intersectionality framework—a framework that considers the simultaneity of different social dimensions—argues that Black women experience multiple chronic stress-related morbidities at a higher rate and earlier than other racial/ethnic-sex groups. To understand if combined ethnoracial, gender and other social position disadvantages amplify the risk of adverse health outcomes, further studies should use the intersectionality framework.^26 31 32^ Furthermore, in the CONSTANCES cohort, the high overweight/obesity rates and the over-representation in the worst DASH tercile (online supplemental table 1)—also influenced by social factors such as structural racism^33 34^—could contribute to explaining the higher BP rates of women of SSA and DROMs, compared with the majority group. These factors were not as marked among their male counterparts, which could partly account for the narrower gender gap in hypertension for these minorities.

Major cross-sectional studies assessing hypertension prevalence and management usually use the same protocol involving three BP measures on the same occasion to assess BP, hypertension, and control; reported hypertension to assess awareness; self-reported hypertension treatment and/or administrative data on hypertension treatment to assess hypertension and treatment.^4 5 35^ These studies use the cascade of care framework founded on the principle that there cannot be treatment without awareness or control without treatment. In our study, however, 8.4% of individuals with high BP do not report hypertension but are considered under hypertensive treatment. This may be due to antihypertensive therapy being used as a preventive treatment for other CVD^36 37^ or in case of prehypertension (130/80 mm Hg<BP<140/90 mm Hg) among patients with a history of other CVD.^2 38 39^ In our hypertensive population, women and men of the SSA have lower pre-existing CVD rates than the majority group (online supplemental table 2), which can partly explain their lower likelihood of being in the ‘Other’ group. However, pre-existing CVD rates do not seem to explain why individuals in the North African group are, on the contrary, more likely than the majority group to be in the ‘Other’ group. Other hypotheses, impossible to test in our dataset, can be put forward. The specific molecules chosen to identify an ‘antihypertensive treatment’ in an administrative dataset (online supplemental table 9) such as the SNDS (*e.g.*, beta blockers) might contribute to a misclassification bias, and a reporting bias might occur at the awareness step (namely hypertensive individuals who do not declare hypertension, because under treatment or controlled). Individuals in the ‘Other’ group could not be considered in the cascade of care as they fell outside the sequential ‘awareness and treatment’ classification.^40^ Some studies have tackled the potential bias caused by this category of respondents by excluding them.^7^ Our study includes these individuals and considers them as a separate group to minimize any bias. To our knowledge, our study is also the only one that uses a multinomial analysis, enabling us to work on a common denominator (*i.e.*, individuals with hypertension) and compare ORs at each step of the cascade.

Men and women from the SSA and DROM groups are less likely than the majority group to remain outside the hypertension care path, and less likely to be in the ‘Other’ path (except for DROM men), resulting in them being more likely to have entered the ‘typical’ hypertension cascade of care (at least by achieving the ‘awareness’ step). The latest European guidelines encourage an increased attention to differences in the management of hypertension across ethnoracial groups, especially regarding individuals with African origins.^2 3^ These guidelines being main contributors to the French Society for Arterial Hypertension, one might assume that these ethnoracial groups are targeted by practitioners in France^41^ to be diagnosed and initiated into care. Individuals of DROMs, especially natives, might also be more targeted by health practitioners in France, as prevalence rates in several DROMs are higher than that in metropolitan France (38.2% in Réunion, 44% in Mayotte).^42^ SSA women, however, are more likely than women of the majority group to remain at the treatment step, rather than reach control, while this result was non-significant for men. This could partly be explained by an overrepresentation of aware and treated SSA women with overweight and obesity (online supplemental table 4), which has been shown to be a challenge for hypertension control.^2 3^ Furthermore, women and immigrants from Africa are more likely to report discrimination in France, which is strongly associated with foregoing care within this ethnoracial group.^30^ The use of the intersectionality framework could clarify if ethnoracial and gender discriminations commingle to produce an amplified effect.^26^ Racism and discrimination can also affect individuals’ relation to the health system and physicians and might lead to therapeutic inertia (*i.e.,* failure to adjust treatment when BP remains above target^2^). Our findings are consistent with results on cohorts from the UK and the Netherlands^8^ suggesting, among other factors, that African individuals might also experience inequalities in access to healthcare compared with the majority group, ultimately impacting control as well.

When it comes to men and women of North African origin, no significant results are found compared with their majority group counterparts regarding their progress through the various stages of the hypertension health cascade of care. This finding is partially supported by a 2010 French clinical study conducted in a hypertension unit, where patients born in North Africa are referred to the unit at similar disease stages (with comparable duration of hypertension, BP levels and treatment scores) as native Europeans.^15^ In our study, the majority of hypertensive individuals of North African origin are second-generation immigrants (online supplemental table 2), a factor known to influence healthcare utilisation towards that of the majority group, largely due to socioeconomic factors.^43^ However, more research is needed, as North African individuals also report significant discrimination rates in a French nationally representative survey in 2008,^30^ which could act as a barrier to access to care^44 45^ and can potentially impact awareness, treatment and control. Men of European and other origin also have a similar cascade of care pattern to the majority group. These men share similar age and health conditions (online supplemental table 2), factors known to be associated with awareness, treatment and control in French studies.^46^,^48^ In contrast, women of European and other origins are more likely than women of the majority group to remain at the awareness or treatment steps rather than achieve control. Further research is needed to explain this result. Lastly, Asian men are more likely than men of the majority group to stay at the awareness step, rather than the control step, while they are as likely to stay at the treatment step. Their higher awareness rates could partly be explained by high education levels in the hypertensive population (online supplemental table 2), a contributor to awareness.^46^ Once aware, their average yearly visit to a GP is lower than the rest of the men (online supplemental table 3), potentially explaining their low treatment level. However, caution is advised in interpreting this result, as the sample sizes were small in some of the Asian subgroups. Further research is also needed to explain why, contrary to their male counterparts, Asian women do not have a different cascade of care profile compared with the majority group.

Overall, our study found that only 12.9% are aware, treated and controlled, which is low compared with other high-income countries.^5^ This can be attributed to several interconnected factors. First, the treatment rate has been declining in recent years among women in France.^49^ This trend is compounded by a notable change in therapeutic approaches: the proportion of patients on monotherapy increased from 45% in 2014 to 55% in 2019, implying a corresponding reduction in the use of combination therapies, which are often more effective for BP control.^50^ Not to mention that fixed-dose triple combinations (*i.e.*, three antihypertensive drugs combined in a single pill), shown to be associated with higher control rates than three separate pills,^51 52^ were not reimbursed in France before 2025, an exception among European countries.^53^ Furthermore, policy changes such as the removal of full health coverage for severe hypertension under the ‘*Affection de Longue Durée*’ (ALD) status—formerly allowing 100% reimbursement for chronic conditions—have reduced access to free care to individuals diagnosed after 2011, possibly impacting control.^53^ Reimbursement is also lacking for home BP monitoring devices, creating financial barriers for optimal management.^54^ Finally, according to a survey on a sample of 753 GPs in France, less than 50% of them reported feeling highly implicated in dietary or overweight and obesity prevention,^55^ often citing lack of time and resources, which further hinders effective BP control.^56^

Our study has some limitations. First, even though the protocol used to describe the hypertension cascade of care has widely been published in international and French studies,^4^,^635 40^ some biases are worth noting. Hypertension may be overestimated, and control underestimated, mainly due to the ‘white coat effect’^57^ when hypertension is measured on the same visit, even if it follows a 3-measure protocol. Awareness is self-reported and can therefore introduce a reporting bias. Treatment is assessed with medication reimbursements, which do not indicate whether the treatment was taken correctly. Non-pharmaceutical recommendations, such as changes to diet/lifestyle, cannot be considered, due to a lack of appropriate data, though such lifestyle changes are an inherent part of the treatment and can even replace pharmaceutical treatment in some specific cases.^2^ This study does not analyse adherence to treatment, while it is instrumental in hypertension long-term care management. Second, even though migratory status has proven to be a valid proxy for race/ethnicity^13^ and has been used in French health research,^58 59^ it is important to emphasize that the two are not equivalent. The use of the latter enables hypotheses regarding racism and ethnoracial discrimination to explain the stated inequalities, although we also lack available data on discrimination.^60^ Furthermore, this approach limits our ability to capture the experiences of third- and fourth-generation individuals, who may no longer be classified as belonging to an ethnoracial minority but continue to face racial discrimination and its associated health impacts.^13 14^ Relying on migratory status also blurs the line between the social experience of migration and that of racialisation and racism. While both can intersect, they represent distinct processes with different social and health consequences.^14^ Third, selection biases limit the representativeness of our sample. The CONSTANCES cohort does not have a HSC in the Seine-Saint-Denis *département* of France, despite it being the poorest department in metropolitan France with the highest concentration of immigrants (39%), descendants of immigrants (28%) and natives and descendants of natives from DROMs (7%).^19^ In addition to cohort participation bias at inclusion, we can also not include some individuals with missing values or whose race/ethnicity was impossible to determine, partly due to the complexity of the French postcolonial context. Lastly, the relatively small sample sizes in some groups limited the robustness of our findings, although we could still identify results and trends in our study.

Overall, our study found differences in hypertension prevalence and management at the intersection of sex and race/ethnicity. Our findings underscore the necessity to take these results into consideration when designing intervention strategies to reduce the burden of uncontrolled hypertension. More generally, our results call for further research on health inequalities using race/ethnicity in France when suitable, as structural factors, such as racial discrimination,^61^ differential access to healthcare and socioeconomic inequalities are likely to impact ethnoracial minorities for other health conditions.

## Supplementary material

10.1136/bmjopen-2024-097800 online supplemental file 1

## Data Availability

Data are available upon reasonable request.
