# Supplementary material for: Prevalence, awareness, treatment and control of hypertension among ethnoracial minorities in France: results from the CONSTANCES cohort
Source: BMJ Open. 2025 Sep 22;15(9):e097800. doi: 10.1136/bmjopen-2024-097800 (PMC12458881; doi:10.1136/bmjopen-2024-097800)
Supplement: online supplemental file 1 [file bmjopen-15-9-s001.docx]

Prevalence, Awareness, Treatment and Control of Hypertension among Ethnoracial Minorities in France: Results from the CONSTANCES Cohort

Supplementary material

[Supplementary Figure 1. Flowchart of the study population 2](#_Toc204867015)

[Supplementary Figure 2. Migration status categorization in CONSTANCES 3](#_Toc204867016)

[Supplementary Table 1. Characteristics the people in the CONSTANCES cohort by race/ethnicity and sex (N=180,459) 4](#_Toc204867017)

[Supplementary Table 2. Characteristics of people with hypertension by race/ethnicity and sex (N=54,009) 5](#_Toc204867018)

[Supplementary Table 3. Characteristics people with hypertension who achieve awareness by race/ethnicity and sex (N=18,999) 6](#_Toc204867019)

[Supplementary Table 4. Characteristics people with hypertension who achieve awareness and treatment by race/ethnicity and sex (N=17,235) 7](#_Toc204867020)

[Supplementary Table 5. Age-standardized hypertension rates by gender and ethnicity (N=54,009) 8](#_Toc204867021)

[Supplementary Table 6. Odds ratio of having hypertension adjusted for age, sex, race/ethnicity and interaction term sex*race/ethnicity (N=180,459) 9](#_Toc204867022)

[Supplementary Table 7a. Odds-ratios of being in different steps of the hypertension care cascade, compared to being controlled among women, according to race/ethnicity and adjusted for age (N=54,009). 10](#_Toc204867023)

[Supplementary Table 7b. Odds-ratios of being in different steps of the hypertension care cascade, compared to being controlled among men, according to race/ethnicity and adjusted for age (N=54,009). 11](#_Toc204867024)

[Supplementary Table 8a. Marginal predicted probabilities by race/ethnicity, from multinomial logistic regression adjusted for age (women) (N=54,009) 12](#_Toc204867025)

[Supplementary Table 8b. Marginal predicted probabilities by race/ethnicity, from multinomial logistic regression adjusted for age (men) (N=54,009) 12](#_Toc204867026)

[Supplementary Table 9. Molecules included in the definition of antihypertensive treatment 13](#_Toc204867027)


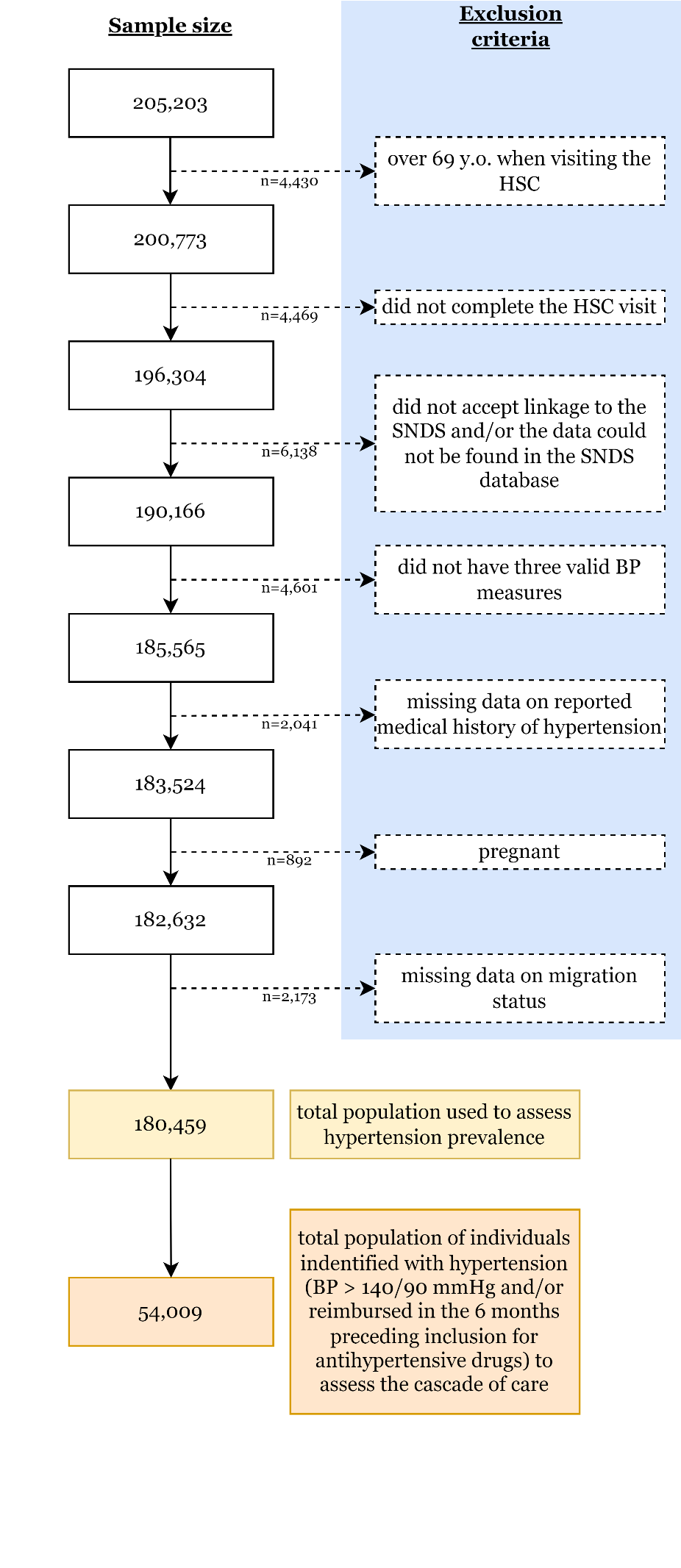


# Supplementary Figure 1. Flowchart of the study population

Notes: HSC, Health Screening Center, SNDS, French National Health Data System, BP, Blood Pressure

**
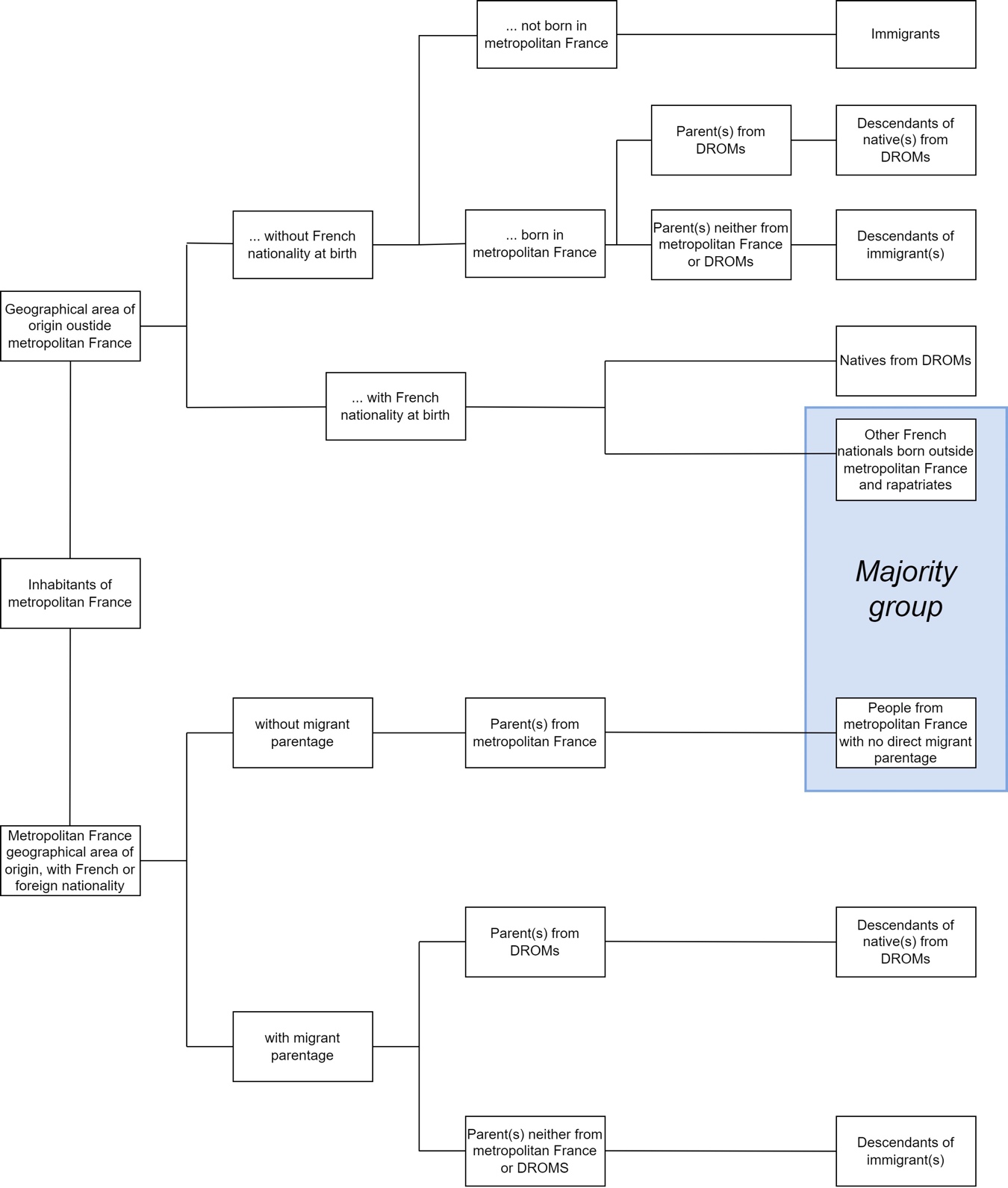
**

# Supplementary Figure 2. Migration status categorization in CONSTANCES

Notes: DROM, French Overseas *départments* and regions, repatriates, French (former settlers, mostly) who left the colonies to settle or resettle in metropolitan France.

For descendants of immigrants (or natives of DROMs), if they are born to a mixed-origin couple, consisting of two migrant parents of different origins, they are assigned the father’s origin, for the sake of simplicity.

# Supplementary Table 1. Characteristics the people in the CONSTANCES cohort by race/ethnicity and sex (N=180,459)

|  | **Women** | | | | | | **Men** | | | | | |
| --- | --- | --- | --- | --- | --- | --- | --- | --- | --- | --- | --- | --- |
|  | **Majority group**  **N=77,121** | **SSA**  **N=1,054** | **DROM**  **N=1,406** | **North Africa**  **N=4,390** | **Europe and Other**  **N=4,390** | **Asia**  **N=4,390** | **Majority group**  **N=68,862** | **SSA**  **N=1,166** | **DROM**  **N=1,056** | **North Africa**  **N=4,385** | **Europe and Other**  **N=4,385** | **Asia**  **N=4,385** |
| **Hypertension** | 23† | 28†* | 23† | 15†* | 22† | 13†* | 39 | 40 | 35* | 29* | 38* | 29* |
| **Age^1^** | 47(13)† | 40(12)†* | 42(13)* | 42(12)†* | 47(13)† | 41(12)†* | 48(13) | 42(12)* | 43(13)* | 44(12)* | 48(13) | 41(13)* |
| **Share of 1st and 2nd generation immigrants** |  |  |  |  |  |  |  |  |  |  |  |  |
| 1st generation |  | 67†* | 67* | 24†* | 38†* | 59†* |  | 77* | 68* | 33* | 33* | 54* |
| 2nd generation |  | 33†* | 33* | 76†* | 62†* | 41†* |  | 23* | 32* | 67* | 67* | 46* |
| **Education** |  |  |  |  |  |  |  |  |  |  |  |  |
| Up to high school | 37† | 50†* | 46* | 38†* | 37†* | 26†* | 42 | 45* | 48* | 43* | 44* | 35* |
| Undergraduate | 40† | 29†* | 32* | 36†* | 34†* | 35†* | 30 | 25* | 29* | 28* | 26* | 26* |
| Postgraduate | 23† | 21†* | 21* | 26†* | 29†* | 39†* | 28 | 30* | 23* | 29* | 30* | 40* |
| **Overweight/**  **obesity** | 35† | 54* | 45†* | 40†* | 36†* | 23†* | 50 | 55* | 53 | 55* | 53* | 38* |
| **Type II Diabetes** | 2.4† | 5.9* | 5.3* | 3.8†* | 2.9†* | 3.9* | 4.9 | 7.8* | 6.9* | 6.7* | 5.8* | 5.6 |
| **Dyslipidemia** | 25† | 16†* | 19†* | 20†* | 25† | 18†* | 38 | 27* | 32* | 38 | 40* | 39 |
| **Other CVD** | 9.3† | 7.0* | 8.3† | 7.5†* | 9.2† | 5.1†* | 14 | 8.3* | 11* | 11* | 14 | 8.0* |
| **Smokes** | 17† | 13†* | 17† | 20†* | 18†* | 15† | 19 | 19* | 21 | 26* | 21* | 24* |
| **Average visits to a GP** | 4.2(3.2)† | 4.3(3.3)† | 4.1(3.2)†* | 4.3(3.6)† | 4.1(3.4)†* | 3.1(2.8)†* | 3.2(2.7) | 3.0(2.7)* | 2.9(2.7)* | 3.4(3.2) | 3.3(3.9) | 2.6(3.0)* |
| **DASH (worst tercile)^1^** | 32† | 44†* | 36†* | 33† | 30†* | 36†* | 55 | 57 | 53 | 50* | 50* | 49* |

^1^ Mean (SD)

† p<0.05 for comparison between men and women within ethnoracial groups (Wilcoxon rank sum test; Pearson's Chi-squared test), * p<0.05 for comparison between each ethnoracial group and their counterparts in the Majority group (for men and women) (Wilcoxon rank sum test; Pearson's Chi-squared test; Fisher's exact test)

Notes: SSA, Subsaharan African group; DROM, Overseas France *départements* and regions; CVD, cardiovascular diseases; GP, General Practioner; DASH, Dietary Approaches to Stop Hypertension

# Supplementary Table 2. Characteristics of people with hypertension by race/ethnicity and sex (N=54,009)

|  | **Women** | | | | | | **Men** | | | | | |
| --- | --- | --- | --- | --- | --- | --- | --- | --- | --- | --- | --- | --- |
|  | **Majority group, N = 17,607** | **SSA, N = 292** | **DROM, N = 324** | **North Africa, N = 668** | **Europe and Others, N = 2,262** | **Asia, N = 166** | **Majority group, N = 27,021** | **SSA, N = 466** | **DROM, N = 373** | **North Africa, N = 1,265** | **Europe and Others,**  **N = 3,302** | **Asia,**  **N = 263** |
| **Age**^1^ | 56(10)† | 50(10)†* | 52(11)† | 52(11)† | 57(10) | 53(11)† | 54(12) | 47(11) | 49(13)* | 50(12) | 55(11) | 50(13) |
| **Share of 1^st^ and 2^nd^ generation immigrants** |  |  |  |  |  |  |  |  |  |  |  |  |
| 1st-generation | - | 84* | 78* | 33* | 36†* | 57* | - | 83* | 75* | 37* | 32* | 56* |
| 2nd-generation | - | 16* | 22* | 67* | 64†* | 43* | - | 17* | 25* | 63* | 68* | 44* |
| **Education level** |  |  |  |  |  |  |  |  |  |  |  |  |
| Up to high school | 53† | 60†* | 63†* | 52* | 54†* | 39* | 52 | 48* | 55 | 50* | 55* | 42* |
| Undergraduate | 34† | 26†* | 25†* | 29* | 29†* | 36* | 27 | 27* | 24 | 26* | 22* | 25* |
| Postgraduate | 13† | 14†* | 12†* | 19* | 17†* | 26* | 21 | 25* | 21 | 23* | 22* | 32* |
| **Overweight/**  **obesity** | 54† | 75* | 67* | 64†* | 58†* | 43†* | 68 | 69 | 68 | 70 | 72* | 62* |
| **Type II Diabetes** | 6.2† | 15* | 13* | 11* | 7.9†* | 8.4 | 10 | 14 | 14* | 13* | 12* | 13 |
| **Dyslipidemia** | 45† | 28†* | 37†* | 40†* | 48†* | 42† | 52 | 36* | 44* | 50 | 55* | 54 |
| **Other CVD** | 18† | 14 | 18 | 16† | 20† | 17 | 24 | 14* | 20* | 24 | 26 | 22 |
| **Smokes** | 11† | 10† | 9.9† | 15†* | 13†* | 12† | 15 | 17 | 16 | 21* | 16* | 21* |
| **Average visits to a GP**^1^ | 5.2(3.7)† | 5.6(4.3)†* | 5.2(3.7)† | 5.6(4.3)† | 5.2(3.8)† | 4.6(4.0)†* | 3.9(3.0) | 3.7(3.1)* | 3.6(3.2)* | 4.2(3.6) | 4.2(5.3)* | 3.8(4.4)* |
| **DASH (worst tercile)** | 30† | 38†* | 32† | 32† | 30† | 37 | 54 | 58 | 51 | 49* | 51* | 45* |

^1^ Mean (SD)

† p<0.05 for comparison between men and women within ethnoracial groups (Wilcoxon rank sum test; Pearson's Chi-squared test), * p<0.05 for comparison between each ethnoracial group and their counterparts in the Majority group (for men and women separately) (Wilcoxon rank sum test; Pearson's Chi-squared test; Fisher's exact test)

Notes: SSA, Subsaharan African group; DROM, Overseas France *départements* and regions group; CVD, cardiovascular diseases; GP, General Practitioner; DASH, Dietary Approaches to Stop Hypertension

# Supplementary Table 3. Characteristics people with hypertension who achieve awareness by race/ethnicity and sex (N=18,999)

|  | **Women** | | | | | | **Men** | | | | | |
| --- | --- | --- | --- | --- | --- | --- | --- | --- | --- | --- | --- | --- |
|  | **Majority group,**  **N = 6,802** | **SSA,**  **N = 154** | **DROM,**  **N = 142** | **North Africa,**  **N = 241** | **Europe and Other,**  **N = 878** | **Asia,**  **N = 72** | **Majority group,**  **N = 8,829** | **SSA,**  **N = 184** | **DROM,**  **N = 122** | **North Africa,**  **N = 360** | **Europe and Other,**  **N = 1,130** | **Asia,**  **N = 85** |
| **Age**^1^ | 59(8)† | 52(9)* | 56(9)* | 56(8)* | 59(8) | 56(9)* | 59(8) | 53(9) | 55(9) | 56(9) | 59(8) | 57(10) |
| **Share of 1st and 2nd generation immigrants** |  |  |  |  |  |  |  |  |  |  |  |  |
| 1st-generation |  | 88* | 89* | 37* | 37 | 60* |  | 90* | 84* | 43* | 34* | 65* |
| 2nd-generation |  | 12* | 11* | 63* | 63 | 40* |  | 10* | 16* | 57* | 66* | 35* |
| **Education level** |  |  |  |  |  |  |  |  |  |  |  |  |
| Up to high school | 60† | 60†* | 68* | 62†* | 59†* | 36* | 58 | 47* | 67* | 51* | 60* | 41* |
| Undergraduate | 30† | 26†* | 20* | 21†* | 28†* | 36* | 24 | 26* | 13* | 26* | 21* | 27* |
| Postgraduate | 9.9† | 14†* | 11* | 17†* | 14†* | 28* | 18 | 27* | 20* | 23* | 20* | 32* |
| **Overweight/**  **obesity** | 64† | 77* | 77* | 73* | 64† | 49†* | 78 | 78 | 84 | 79 | 82* | 72 |
| **Type II Diabetes** | 10† | 19* | 18†* | 18* | 12† |  | 18 | 22 | 29* | 22 | 21* | 19 |
| **Dyslipidemia** | 53† | 35†* | 39†* | 46†* | 53† | 47 | 65 | 48* | 55* | 64 | 66 | 62* |
| **Other CVD** | 22† | 18 | 25 | 19† | 24† | 18† | 36 | 23* | 35 | 36 | 36 | 32* |
| **Smokes** | 10† | 9.1 | 9.2 | 15* | 12 | 14 | 12 | 15 | 11 | 19* | 13 | 24 |
| **Average visits to a GP**^1^ | 6.2(3.7)† | 6.4(4.0)† | 5.8(3.6) | 6.9(5.1)† | 6.1(3.8)† | 5.0(3.3)* | 5.2(3.1) | 4.8(3.4)* | 5.5(3.8) | 5.5(3.5) | 5.4(3.5) | 4.6(5.2)* |
| **DASH (worst tercile)** | 30† | 34† | 30† | 29† | 29† | 28† | 52 | 53 | 52 | 52 | 52 | 46 |

^1^ Mean (SD)

† p<0.05 for comparison between men and women within ethnoracial groups (Wilcoxon rank sum test; Pearson's Chi-squared test), * p<0.05 for comparison between each ethnoracial group and their counterparts in the Majority group (for men and women separately) (Wilcoxon rank sum test; Pearson's Chi-squared test; Fisher's exact test)

Notes: SSA, Subsaharan African group; DROM, Overseas France *départements* and regions group; CVD, cardiovascular diseases; GP, General Practioner; DASH, Dietary Approaches to Stop Hypertension

# Supplementary Table 4. Characteristics people with hypertension who achieve awareness and treatment by race/ethnicity and sex (N=17,235)

|  | **Women** | | | | | | **Men** | | | | | |
| --- | --- | --- | --- | --- | --- | --- | --- | --- | --- | --- | --- | --- |
|  | **Majority group,**  **N = 6,244** | **SSA,**  **N = 139** | **DROM,**  **N = 124** | **North Africa,**  **N = 210** | **Europe and Other,**  **N = 784** | **Asia,**  **N = 61** | **Majority group,**  **N = 8,023** | **SSA,**  **N = 157** | **DROM, N = 102** | **North Africa, N = 309** | **Europe and Other,**  **N = 1,016** | **Asia,**  **N = 66** |
| **Age**^1^ | 59(8)† | 53(9)* | 56(9)* | 57(8)* | 60(8) | 58(7)* | 60(8) | 53(9)* | 56(8)* | 57(8)* | 60(8) | 58(9) |
| **Share of 1st and 2nd generation immigrants** |  |  |  |  |  |  |  |  |  |  |  |  |
| 1st-generation |  | 87* | 90* | 36* | 37* | 61* |  | 91* | 82* | 43* | 34* | 65* |
| 2nd-generation |  | 13* | 10* | 64* | 63* | 39* |  | 8.9* | 18* | 57* | 66* | 35* |
| **Education level** |  |  |  |  |  |  |  |  |  |  |  |  |
| Up to high school | 61† | 59† | 70 | 66†* | 60†* | 39* | 59 | 47* | 67 | 50* | 61* | 42* |
| Undergraduate | 30† | 27† | 20 | 20†* | 27†* | 34* | 24 | 25* | 15 | 26* | 20* | 26* |
| Postgraduate | 9.5† | 14† | 9.7 | 14†* | 13†* | 26* | 17 | 27* | 19 | 24* | 19* | 32* |
| **Overweight/**  **obesity** | 65† | 79* | 79* | 73†* | 65† | 49†* | 79 | 78 | 82 | 80 | 83* | 74 |
| **Type II Diabetes** | 10† | 19* | 20†* | 19* | 12† |  | 19 | 24 | 34* | 25* | 22* | 21 |
| **Dyslipidemia** | 54† | 35†* | 40* | 48† | 55† | 52† | 66 | 51 | 60 | 66 | 67 | 70 |
| **Other CVD** | 22† | 19 | 25† | 20† | 24† | 20 | 38 | 25 | 36 | 38 | 38 | 38 |
| **Smokes** | 10† | 9.4 | 9.7 | 14* | 11 |  | 12 | 13* | 12 | 17* | 12 | 24* |
| **Average visits to a GP**^1^ | 6.3(3.7)† | 6.8(3.9)† | 6.1(3.4) | 7.2(5.2)† | 6.2(3.8)† | 5.3(3.1)* | 5.4(3.1) | 5.2(3.4) | 5.8(3.7) | 5.8(3.6)* | 5.6(3.5) | 5.2(5.7)* |
| **DASH (worst tercile)** | 29† | 32† | 31† | 29† | 29† | 25† | 52 | 55 | 53 | 51 | 52 | 48 |

^1^ Mean (SD)

† p<0.05 for comparison between men and women within ethnoracial groups (Wilcoxon rank sum test; Pearson's Chi-squared test), * p<0.05 for comparison between each ethnoracial group and their counterparts in the Majority group (for men and women separately) (Wilcoxon rank sum test; Pearson's Chi-squared test; Fisher's exact test)

Notes: SSA, Subsaharan African group; DROM, Overseas France *départements* and regions group; CVD, cardiovascular diseases; GP, General Practioner; DASH, Dietary Approaches to Stop Hypertension. Some cells were left empty for confidentiality reasons.

# Supplementary Table 5. Age-standardized hypertension rates by gender and ethnicity (N=54,009)

| **Characteristics** | **Age-standardized hypertension prevalence rates** |
| --- | --- |
| **Sex** |  |
| Women | 20.87 |
| Men | 35.39 |
| **Race/ethnicity** |  |
| Majority group | 27.78 |
| SSA | 40.33 |
| DROM | 31.62 |
| North Africa | 25.29 |
| Europe and other | 26.92 |
| Asia | 25.14 |

Notes: SSA, Subsaharan African group; DROM, Overseas France *départements* and regions group

# Supplementary Table 6. Odds ratio of having hypertension adjusted for age, sex, race/ethnicity and interaction term sex*race/ethnicity (N=180,459)

| **Characteristics** | **OR** | **95% CI** | | **p-value** |
| --- | --- | --- | --- | --- |
| **Race/ethnicity** |  |  |  |  |
| Majority group (ref) | — | — | — |  |
| SSA | 1.64 | 1.44 | 1.87 | <0.001 |
| DROM | 1.25 | 1.08 | 1.43 | 0.002 |
| North Africa | 0.81 | 0.75 | 0.87 | <0.001 |
| Europe and other | 0.94 | 0.89 | 0.99 | 0.021 |
| Asia | 0.94 | 0.80 | 1.10 | 0.5 |
| **Sex** |  |  |  |  |
| Men (ref) | — | — | — |  |
| Women | 0.42 | 0.41 | 0.43 | <0.001 |
| **Age** | 1.08 | 1.08 | 1.08 | <0.001 |
| **Race/ethnicity*Sex** |  |  |  |  |
| SSA | 1.52 | 1.25 | 1.84 | <0.001 |
| DROM | 1.21 | 1.00 | 1.48 | 0.053 |
| North Africa | 1.03 | 0.92 | 1.15 | 0.6 |
| Europe and other | 1.04 | 0.97 | 1.12 | 0.2 |
| Asia | 0.81 | 0.64 | 1.03 | 0.089 |

Notes: SSA, Subsaharan African group; DROM, Overseas France départements and regions group; OR, Odds Ratio; 95%CI, 95% Confidence Interval

# Supplementary Table 7a. Odds-ratios of being in different steps of the hypertension care cascade, compared to being controlled among women, according to race/ethnicity and adjusted for age (N=54,009).

|  | **Not included in a hypertension care path** | | | **Awareness** | | | **Treatment** | | | **Other** | | |
| --- | --- | --- | --- | --- | --- | --- | --- | --- | --- | --- | --- | --- |
|  | **OR***^1^* | **95% CI***^1^* | **p-value** | **OR***^1^* | **95% CI***^1^* | **p-value** | **OR***^1^* | **95% CI***^1^* | **p-value** | **OR***^1^* | **95% CI***^1^* | **p-value** |
| **Race/ethnicity** |  |  |  |  |  |  |  |  |  |  |  |  |
| Majority group (ref) | — | — |  | — | — |  | — | — |  | — | — |  |
| SSA | 0.48 | 0.35, 0.66 | <0.001 | 1.01 | 0.57, 1.80 | >0.9 | 1.39 | 0.99, 1.96 | 0.059 | 0.38 | 0.24, 0.62 | <0.001 |
| DROM | 0.74 | 0.54, 1.01 | 0.055 | 1.45 | 0.84, 2.48 | 0.2 | 1.19 | 0.83, 1.70 | 0.3 | 0.70 | 0.46, 1.07 | 0.10 |
| North Africa | 0.84 | 0.67, 1.04 | 0.12 | 1.33 | 0.88, 2.01 | 0.2 | 0.92 | 0.69, 1.21 | 0.5 | 1.27 | 0.97, 1.66 | 0.081 |
| Europe and Other | 1.10 | 0.97, 1.25 | 0.13 | 1.46 | 1.14, 1.87 | 0.002 | 1.15 | 0.99, 1.33 | 0.075 | 1.17 | 0.99, 1.39 | 0.069 |
| Asia | 0.77 | 0.50, 1.19 | 0.2 | 1.82 | 0.91, 3.67 | 0.092 | 1.07 | 0.65, 1.78 | 0.8 | 0.84 | 0.47, 1.50 | 0.6 |
| **Age** | 0.97 | 0.97, 0.97 | <0.001 | 0.97 | 0.96, 0.98 | <0.001 | 1.03 | 1.02, 1.03 | <0.001 | 0.94 | 0.94, 0.95 | <0.001 |
| *^1^* OR = Odds Ratio, CI = Confidence Interval  Notes: SSA, Subsaharan African group; DROM, Overseas France *départements* and regions group | | | | | | | | | | | | |

# Supplementary Table 7b. Odds-ratios of being in different steps of the hypertension care cascade, compared to being controlled among men, according to race/ethnicity and adjusted for age (N=54,009).

|  | **Not included in a hypertension care path** | | | **Awareness** | | | **Treatment** | | | **Other** | | |
| --- | --- | --- | --- | --- | --- | --- | --- | --- | --- | --- | --- | --- |
|  | **OR***^1^* | **95% CI***^1^* | **p-value** | **OR***^1^* | **95% CI***^1^* | **p-value** | **OR***^1^* | **95% CI***^1^* | **p-value** | **OR***^1^* | **95% CI***^1^* | **p-value** |
| **Race/ethnicity** |  |  |  |  |  |  |  |  |  |  |  |  |
| Majority group (ref) | — | — |  | — | — |  | — | — |  | — | — |  |
| SSA | 0.50 | 0.36, 0.68 | <0.001 | 1.17 | 0.73, 1.89 | 0.5 | 1.19 | 0.85, 1.66 | 0.3 | 0.44 | 0.25, 0.77 | 0.004 |
| DROM | 0.66 | 0.47, 0.93 | 0.019 | 1.28 | 0.74, 2.21 | 0.4 | 0.86 | 0.58, 1.28 | 0.5 | 0.64 | 0.37, 1.13 | 0.12 |
| North Africa | 0.90 | 0.73, 1.10 | 0.3 | 1.24 | 0.88, 1.74 | 0.2 | 0.92 | 0.72, 1.16 | 0.5 | 1.33 | 1.01, 1.75 | 0.040 |
| Europe and Other | 0.95 | 0.84, 1.07 | 0.4 | 1.14 | 0.91, 1.43 | 0.3 | 1.01 | 0.88, 1.16 | 0.9 | 1.16 | 0.97, 1.38 | 0.10 |
| Asia | 0.71 | 0.47, 1.07 | 0.10 | 1.85 | 1.03, 3.33 | 0.041 | 0.69 | 0.42, 1.12 | 0.13 | 0.92 | 0.51, 1.67 | 0.8 |
| **Age** | 0.93 | 0.93, 0.94 | <0.001 | 0.95 | 0.94, 0.95 | <0.001 | 1.02 | 1.02, 1.03 | <0.001 | 0.98 | 0.97, 0.98 | <0.001 |
| *^1^* OR = Odds Ratio, CI = Confidence Interval  Notes: SSA, Subsaharan African group; DROM, Overseas France *départements* and regions group | | | | | | | | | | | | |

# Supplementary Table 8a. Marginal predicted probabilities by race/ethnicity, from multinomial logistic regression adjusted for age (women) (N=54,009)

|  | **Not included in a hypertension care path** | **Awareness** | **Treatment** | **Control** | **Other** |
| --- | --- | --- | --- | --- | --- |
| **Majority group** | 51.8% (51.0-52.6) | 3.3% (3.0-3.6) | 16.6% (15.9-17.2) | 17.5% (16.9-18.1) | 10.9% (10.4-11.4) |
| **SSA** | 34.1% (28.4-39.8) | 4.6% (2.2-6.9) | 31.6% (25.5-37.7) | 24.0% (18.7-29.4) | 5.7% (3.4-8.1) |
| **DROM** | 43.6% (37.9-49.3) | 5.4% (2.8-7.9) | 22.4% (17.3-27.4) | 19.9% (15.2-24.7) | 8.7% (5.8-11.6) |
| **North Africa** | 46.0% (42.0-50.0) | 4.6% (3.0-6.3) | 16.1% (13.0-19.2) | 18.6% (15.4-21.8) | 14.7% (12.0-17.4) |
| **Europe and Other** | 51.4% (49.2-53.5) | 4.3% (3.4-5.2) | 17.1% (15.5-18.7) | 15.8% (14.2-17.3) | 11.5% (10.1-12.9) |
| **Asia** | 44.3% (36.3-52.3) | 6.6% (2.7-10.6) | 19.6% (13.0-26.2) | 19.3% (12.9-25.8) | 10.1% (5.6-14.7) |

Notes: SSA, Subsaharan African group; DROM, Overseas France *départements* and regions group

Marginal predicted probabilities for each hypertension outcome category, by race/ethnicity. Estimates are derived from the corresponding multinomial logistic regression model adjusted for age and represent the average predicted probability of each outcome for individuals of each ethnoracial group, holding age constant via marginal standardization.

Each cell shows the predicted probability (%) of each outcome category for women of each racial group, adjusted for age, with 95% confidence intervals in parentheses. *For example, among women in the SSA group, the predicted probability of not being included in a hypertension care path is 34.1% (28.4-39.8%), the predicted probability of being aware (not treated or controlled) is 4.6% (2.2-6.9%), the predicted probability of being (aware and) treated (not controlled) is 31.6% (25.5-37.7%), the predicted probability of being (aware, treated, and) controlled is 24% (18.7-29.4%), the predicted probability of being in another care path is 5.7% (3.4-8.1%).*

# Supplementary Table 8b. Marginal predicted probabilities by race/ethnicity, from multinomial logistic regression adjusted for age (men) (N=54,009)

|  | **Not included in a hypertension care path** | **Awareness** | **Treatment** | **Control** | **Other** |
| --- | --- | --- | --- | --- | --- |
| **Majority group** | 64.4% (63.8-65.1) | 3.2% (3.0-3.5) | 16.4% (15.9-16.9) | 9.4% (9.0-9.8) | 6.5% (6.2-6.9) |
| **SSA** | 47.4% (42.2-52.5) | 5.6% (3.4-7.8) | 28.8% (23.9-33.7) | 14.0% (10.3-17.6) | 4.3% (2.2-6.3) |
| **DROM** | 57.2% (51.4-62.9) | 5.6% (3.1-8.0) | 18.9% (14.4-23.5) | 12.7% (8.8-16.5) | 5.6% (3.0-8.3) |
| **North Africa** | 60.9% (57.9-63.9) | 4.2% (3.0-5.4) | 15.8% (13.6-18.0) | 9.9% (8.1-11.8) | 9.2% (7.4-10.9) |
| **Europe and other** | 62.1% (60.3-63.9) | 3.7% (3.0-4.4) | 16.9% (15.5-18.2) | 9.6% (8.6-10.6) | 7.7% (6.7-8.7) |
| **Asia** | 58.4% (51.7-65.1) | 7.6% (4.2-11.0) | 14.3% (9.7-18.9) | 12.0% (7.7-16.4) | 7.7% (4.1-11.2) |

Notes: SSA, Subsaharan African group; DROM, Overseas France *départements* and regions group

Marginal predicted probabilities for each hypertension outcome category, by race/ethnicity. Estimates are derived from the corresponding multinomial logistic regression model adjusted for age and represent the average predicted probability of each outcome for individuals of each ethnoracial group, holding age constant via marginal standardization.

Each cell shows the predicted probability (%) of each outcome category for men of each racial group, adjusted for age, with 95% confidence intervals in parentheses. *For example, among men in the SSA group, the predicted probability of not being included in a hypertension care path is 47.4% (42.2-52.5%), the predicted probability of being aware (not treated or controlled) is 5.6% (3.4-7.8%), the predicted probability of being (aware and) treated (not controlled) is 28.8% (23.9-33.7%), the predicted probability of being (aware, treated, and) controlled is 14% (10.3-17.6%), the predicted probability of being in another care path is 4.3% (2.2-6.3%).*

| ● **ANTIHYPERTENSIVES**  **ANTIADRENERGIC AGENTS, CENTRALLY ACTING**: C02AB02 (methyldopa (racemic)), C02AC01 (clonidine), C02AC02 (guanfacine), C02AC05 (moxonidine), C02AC06 (rilmenidine).  **ARTERIOLAR SMOOTH MUSCLE, AGENTS ACTING ON**: C02DC01 (minoxidil). **ANTIHYPERTENSIVES AND DIURETICS IN COMBINATION**: C02LA01 (reserpine and diuretics).  ● **DIURETICS**  **LOW-CEILING DIURETICS, THIAZIDES**: C03AA01 (bendroflumethiazide), C03AA03 (hydrochlorothiazide).  **LOW-CEILING DIURETICS, EXCLUDING THIAZIDES**: C03BA04 (chlortalidone), C03BA10 (xipamide), C03BA11 (indapamide), C03BX03 (cicletanine).  **HIGH-CEILING DIURETICS**: C03CA01 (furosemide), C03CA02 (bumetanide), C03CA03 (piretanide). **ALDOSTERONE ANTAGONISTS AND OTHER POTASSIUM-SPARING AGENTS**: C03DA01 (spironolactone), C03DB01 (amiloride).  **DIURETICS AND POTASSIUM-SPARING AGENTS IN COMBINATION:** C03EA01 (hydrochlorothiazide and potassium-sparing agents), C03EA04 (altizide and potassium-sparing agents).  ● **BETA-BLOCKERS**  **BETA BLOCKING AGENTS**: C07AA02 (oxprenolol), C07AA03 (pindolol), C07AA05 (propranolol), C07AA06 (timolol), C07AA12 (nadolol), C07AA15 (carteolol), C07AA16 (tertatolol), C07AA23 (penbutolol), C07AB02 (metoprolol), C07AB03 (atenolol), C07AB04 (acebutolol), C07AB05 (betaxolol), C07AB07 (bisoprolol), C07AB08 (celiprolol), C07AB12 (nebivolol), C07AG01 (labetalol).  **BETA BLOCKING AGENTS AND THIAZIDES:** C07BA02 (oxprenolol and thiazides), C07BB02 (metoprolol and thiazides), C07BB03 (atenolol and thiazides), C07BB07 (bisoprolol and thiazides), C07BB12 (nebivolol and thiazides).  **BETA BLOCKING AGENTS AND OTHER DIURETICS**: C07CA03 (pindolol and other diuretics).  **BETA BLOCKING AGENTS, THIAZIDES AND OTHER DIURETICS**: C07DA06 (timolol, thiazides and other diuretics).  **BETA BLOCKING AGENTS, OTHER COMBINATIONS**: C07FB02 (metoprolol and felodipine), C07FB03 (atenolol and nifedipine).  ● **CALCIUM CHANNEL BLOCKERS**  **SELECTIVE CALCIUM CHANNEL BLOCKERS WITH MAINLY VASCULAR EFFECTS**: C08CA01 (amlodipine), C08CA02 (felodipine), C08CA03 (isradipine), C08CA04 (nicardipine), C08CA05 (nifedipine), C08CA08 (nitrendipine), C08CA09 (lacidipine), C08CA11 (manidipine), C08CA13 (lercanidipine), C08CX01 (mibefradil).  **SELECTIVE CALCIUM CHANNEL BLOCKERS WITH DIRECT CARDIAC EFFECTS**: C08DA01 (verapamil), C08DB01 (diltiazem).  **CALCIUM CHANNEL BLOCKERS AND DIURETICS**: C08GA02 (amlodipine and diuretics).  ● **DRUGS TARGETING THE RENIN-ANGIOTENSIN SYSTEM**  **ACE INHIBITORS, PLAIN**: C09AA01 (captopril), C09AA02 (enalapril), C09AA03 (lisinopril), C09AA04 (perindopril), C09AA05 (ramipril), C09AA06 (quinapril), C09AA07 (benazepril), C09AA08 (cilazapril), C09AA09 (fosinopril), C09AA10 (trandolapril), C09AA13 (moexipril), C09AA15 (zofenopril), C09AA16 (imidapril).  **ACE INHIBITORS, COMBINATIONS**: C09BA01 (captopril and diuretics), C09BA02 (enalapril and diuretics), C09BA03 (lisinopril and diuretics), C09BA04 (perindopril and diuretics), C09BA05 (ramipril and diuretics), C09BA06 (quinapril and diuretics), C09BA07 (benazepril and diuretics), C09BA09 (fosinopril and diuretics), C09BA15 (zofenopril and diuretics), C09BB02 (enalapril and lercanidipine), C09BB04 (perindopril and amlodipine), C09BB10 (trandolapril and verapamil), C09BX02 (perindopril and bisoprolol).  **ANGIOTENSIN II RECEPTOR BLOCKERS (ARBs), PLAIN**: C09CA01 (losartan), C09CA02 (eprosartan), C09CA03 (valsartan), C09CA04 (irbesartan), C09CA06 (candesartan), C09CA07 (telmisartan), C09CA08 (olmesartan medoxomil).  **ANGIOTENSIN II RECEPTOR BLOCKERS (ARBs), COMBINATIONS**: C09DA01 (losartan and diuretics), C09DA02 (eprosartan and diuretics), C09DA03 (valsartan and diuretics), C09DA04 (irbesartan and diuretics), C09DA06 (candesartan and diuretics), C09DA07 (telmisartan and diuretics), C09DA08 (olmesartan medoxomil and diuretics), C09DB01 (valsartan and amlodipine), C09DB02 (olmesartan medoxomil and amlodipine), C09DB04 (telmisartan and amlodipine).  **OTHER AGENTS ACTING ON THE RENIN-ANGIOTENSIN SYSTEM**: C09XA02 (aliskiren), C09XA52  (aliskiren and hydrochlorothiazide).  ● **DRUGS TARGETING THE RENIN-ANGIOTENSIN SYSTEM**  **LIPID MODIFYING AGENTS IN COMBINATION WITH CHANNEL BLOCKERS:** C10BX03 (atorvasatin and amlodipine) |
| --- |

# Supplementary Table 9. Molecules included in the definition of antihypertensive treatment
