# Supplementary material for: Prevalence, awareness, treatment and control of hypertension among ethnoracial minorities in France: results from the CONSTANCES cohort
Source: BMJ Open. 2025 Sep 22;15(9):e097800. doi: 10.1136/bmjopen-2024-097800 (PMC12458881; doi:10.1136/bmjopen-2024-097800)
Supplement: Supplementary file 2 [file bmjopen-2024-097800.reviewer_comments.pdf]

# PEER REVIEW HISTORY

BMJ Open publishes all reviews undertaken for accepted manuscripts. Reviewers are asked to complete a checklist review form and are provided with free text boxes to elaborate on their assessment. These free text comments are reproduced below.

## ARTICLE DETAILS

### Title (Provisional)

Prevalence, Awareness, Treatment and Control of Hypertension among Ethnoracial Minorities in France: Results from the CONSTANCES Cohort

### Authors

Silberzan, Léna; Wiernik, Emmanuel; Bajos, Nathalie; Kelly-Irving, Michelle

---

## VERSION 1 - REVIEW

---

| | |
|---|---|
| **Reviewer** | **1** |
| **Name** | **Vriend, Esther** |
| **Affiliation** | **University of Amsterdam** |
| **Date** | **02-May-2025** |
| **COI** | **None** |

---

This paper investigates ethnic differences in the prevalence of hypertension within the French CONSTANCES cohort. It is a relevant and interesting topic. However, several major revisions are needed to improve clarity and readability.

Overall:

- The categorization of ethnic groups lacks clarity. Using "the majority" as an ethnic group is not appropriate. Consider rephrasing to "individuals of European descent" . Additionally, the category "European and other" needs further explanation.

- The analysis is limited to age-adjusted models stratified by sex. Additional models adjusting for confounders such as BMI, other cardiovascular risk factors, and socioeconomic status are necessary to better understand the observed differences.

- The use of “steps” is unnecessarily complex, especially the undefined “other” step. Consider presenting results for awareness, treatment, and control as separate outcomes.

Abstract:

- Include the number of women in the cohort and provide percentages for each ethnic group.

- Add the mean age of participants.

Introduction:

The introduction is currently too long and could benefit from a more concise, focused presentation.

Methods:

- The definition of ethnicity is unclear. While migration status is used as a proxy, it is important to clarify how individuals of mixed ethnic background were categorized and how it is based on the migration status of their parents.

- Please clarify what is meant by “overseas France,” as this term encompasses a range of regions and populations.

- How were discrepancies between self-reported data and registry data handled? For instance, history of cardiovascular disease was assessed through both self-report and the SNDS.

- Please add a participant flowchart (as supplementary material) to clarify the selection process.

Results:

- Please begin the Results section with descriptive statistics: mean age, number of women, and the ethnic distribution of the cohort.

- The “other” step is not clearly defined and lacks clinical relevance. It may be better to omit this and present the outcomes (awareness, treatment, control) separately.

Tables and Figures:

- Table 1: Add the total number of participants in each category.

- For Figures 3 and 4, add brief explanations of the steps underneath the figures for reader clarity.

- As noted earlier, consider removing the “other” step, especially since Figure 1 notes that it is not compatible with clinical interpretation.

Discussion:

- The discussion focuses on comparisons with studies from the USA. Please consider referencing to European studies (the HELIUS study for example), as population characteristics of European countries are more comparable.

- Similar to the introduction, the discussion is too long, please discuss your results more to-the-point.

- Provide an explanation why such a small proportion of individuals are treated, aware, or in control of their hypertension (only 12.9%).

---

**Reviewer 2**

**Name** **Jiao, Lirui**

**Affiliation** **The University of North Carolina at Chapel Hill Gillings School of Global Public Health**

**Date** **20-Jun-2025**

**COI** **None**

---

This study investigates the intersection of sex and ethnicity in hypertension prevalence and care outcomes in France, using data from over 180,000 adults in the CONSTANCES cohort linked to national health records. By using migration status as a proxy for ethnicity, the authors examined age-standardized hypertension prevalence and progression through the care cascade (i.e., awareness, treatment, and control) via multinomial logistic regression. The findings reveal significant disparities, where individuals from Sub-Saharan Africa and Overseas France, particularly women, have higher hypertension prevalence and are more likely to remain in treatment without achieving control. Other groups, such as women of European/other origin and Asian men, are more likely to remain in the aware step. The study highlights the importance of tailoring hypertension interventions to both ethnic and sex-specific patterns.

Introduction

The introduction of the study provides a strong rationale for the research by clearly establishing hypertension as a significant public health concern globally. It effectively contextualizes the importance of examining disparities across the care cascade and frames ethnicity, examined using migration status as a proxy, as a key yet underexplored determinant in France. The authors provide a comprehensive overview of literature from the U.S. and other European countries and emphasize the lack of comparable data in France due to legal constraints on collecting ethnic data.

1. The last sentence in the third paragraph (Line 55-56) contains errors and need to be revised for clarity.

2. The authors could state more explicitly the contribution of the present study relative to prior French studies, beyond simply addressing a gap in the literature.

3. In addition, while the authors note that only two clinical studies have examined hypertension by migration status, it is unclear how this study expands upon those studies.

4. The authors do a good job of explaining why ethnic data is difficult to obtain, however, they should provide further context on the current proportions of the ethnicity groups among the French population and explicate more clearly why migration status is a reasonable proxy.

Methods

The use of the CONSTANCES cohort enhances generalizability to the French adult population aged 18–69. The care cascade logic (control implies treatment, treatment implies awareness) is clearly articulated and consistent with prior literature. Appropriate statistical approaches were also used. However, there are a few weaknesses that require attention.

1. The authors should discuss the limitations of using migration status as an imprecise proxy for ethnicity in this section. It would also be helpful to cite prior studies that have used this approach if possible.

2. Although education is included, the authors should consider also controlling for other socioeconomic variables, such as income, employment, etc, to address confoundedness.

3. The authors could consider adding a flow chart to show how they obtained their final sample.

Results

The results section presents a comprehensive and well-structured summary of findings, with clear reporting of descriptive statistics and regression estimates, stratified by sex and ethnic groups. However, some areas could be improved to strengthen interpretability and rigor.

1. While reporting odds ratios is appropriate for multinomial logistic regression, the use of odds ratio limits interpretability for the audience. The authors could improve upon this by acknowledging the limitations of interpreting ORs as risks, especially when outcomes are common, and by providing additional estimates (e.g., marginal probabilities or absolute differences) to enhance interpretability and policy relevance.

2. The authors should clarify whether education, overweight/obesity, or other covariates were included in sensitivity models. The results do not report whether odds ratios differ significantly across subgroups beyond sex (e.g., interaction terms by migration group and education level).

Discussion

The discussion section offers a thoughtful and well-contextualized interpretation of the study's findings. However, the authors should revise their current sentence "These studies show that individuals of African origin in Europe (resp. African Americans in the US) consistently have higher hypertension rates, putting forward biological hypotheses, such as early arterial stiffening, higher salt-sensitivity and propension to overweight and obesity" to avoid implying a biological determinism linked to ethnicity.

Overall, this study provides a large-scale and timely examination of the hypertension care cascade by sex and ethnicity, using a large, population-based French cohort. However, revisions are needed to improve clarity and interpretability in the method and results sections, and authors should take caution to not conflate ethnicity with biological constructs in the discussion section. Furthermore, authors should check for grammatical errors throughout the manuscript.

## VERSION 1 - AUTHOR RESPONSE

Reviewer: 1
Ms. Esther Vriend, University of Amsterdam
Comments to the Author:
This paper investigates ethnic differences in the prevalence of hypertension within the French CONSTANCES cohort. It is a relevant and interesting topic. However, several major revisions are needed to improve clarity and readability.

**We would like to thank Dr Vriend for the time and effort put in reviewing this article. Please find below the responses to the comments.**

Overall:
- The categorization of ethnic groups lacks clarity. Using "the majority" as an ethnic group is not appropriate. Consider rephrasing to "individuals of European descent" . Additionally, the category "European and other" needs further explanation.

**To better explain how the categorization algorithm we use works, we added Supplementary Figure 1 (Migration status categorization in CONSTANCES) in the Supplementary material.**

**Furthermore, we added information in the methods section to provide further theorical and practical information on how we coded migration status, used as a proxy for race/ethnicity:**

**"Migration status was used as a proxy for race/ethnicity and was assessed using the participant's response to questions about their parents' and their own geographical area of origin (eight item question: metropolitan France/DROM/Europe/North Africa/Subsaharan Africa/Asia/Other/cannot answer), and nationality (Supp Fig 1). Doing so, the "majority group" was defined as all individuals residing in metropolitan France who are neither immigrants, nor DROMs natives (DROMs consists of French territories located outside Europe, largely made up of the remaining parts of the former French colonial empire that continued to be integrated into the French state under different legal statuses after decolonization)[1], nor descendants of these populations. The term "majority group" captures both a statistical overrepresentation, as well as an advantaged position within the stratified structure of French society [19] (in contrast, group sizes are smaller in the other migratory status groups, that can be referred to as "minorities", and individuals in these groups are treated differently in France with respect to their origin[19]). The experience of discrimination and racism more**

[1] Among these territories, the most populated ones (over 200,000 inhabitants in 2021) are Guadeloupe, Martinique, French Guiana, Réunion, Mayotte, French Polynesia, and New Caledonia

**directly concerns immigrants and descendants from North Africa, Subsaharan Africa (SSA), Asia, along with DROMs natives and descendants[19]. For this reason, along with statistical reasons, immigrants and descendants of immigrants from the same geographical area of origin were grouped together, as well as DROMs natives and descendants[19] (Europe and other geographical origin were also grouped together, as it likely that they experience less direct discriminations linked to racism and xenophobia). Migration status, used as a proxy for race/ethnicity was therefore coded as follows: majority group, Sub Saharan African (SSA) group, DROMs group, North African group, Europe and others group, Asian group."**

**Added reference:**

[19] Beauchemin, Cris, Christelle Hamel, et Patrick Simon, éd. *Trajectories and Origins: Survey on the Diversity of the French Population*. Vol. 8. INED Population Studies. Cham: Springer International Publishing, 2018. https://doi.org/10.1007/978-3-319-76638-6.

**This paragraph adds information to how we can consider "majority group" as an ethnoracial category, and why using this definition, we cannot name it "individuals with European descent".**

- The analysis is limited to age-adjusted models stratified by sex. Additional models adjusting for confounders such as BMI, other cardiovascular risk factors, and socioeconomic status are necessary to better understand the observed differences.

**We acknowledge the reviewer's comment regarding checking for potential confounders. We evaluated several candidate variables for potential confounding, including (BMI, other CVD, other socioeconomic position), but none met the necessary criteria of being associated with both the exposure and the outcome without being on the causal pathway. As such, no confounders were identified.**

**The primary objective of our study was to provide a descriptive overview of the observed differences, rather than to investigate the underlying mechanisms. While we agree that further adjustment could offer additional insights, such analyses fall outside the intended scope of this work.**

**We added a sentence in the methods section: "As the objective of our study was to provide a descriptive overview of the distribution prevalence of hypertension and each stage of the hypertension care cascade at the intersection of sex, age, and race/ethnicity, we did not investigate causal mechanisms. Therefore, we did not adjust for other variables than age and race/ethnicity, to avoid overadjustment bias [21]."**

**Added reference:**

[19] Zwieten, Anita van, Peter W. G. Tennant, Michelle Kelly-Irving, Fiona M. Blyth, Armando Teixeira-Pinto, et Saman Khalatbari-Soltani. « Avoiding overadjustment bias in social epidemiology through appropriate covariate selection: a primer ». *Journal of Clinical Epidemiology* 149 (1 septembre 2022): 127-36. https://doi.org/10.1016/j.jclinepi.2022.05.021.

- The use of "steps" is unnecessarily complex, especially the undefined "other" step. Consider presenting results for awareness, treatment, and control as separate outcomes.

**We agree that the use of "steps" affects the readability and clarity of the manuscript. We renamed the steps as follows: Step 0: "not in a hypertension path"; Step 1: "awareness"; Step 2: "treatment"; Step 3: "control"; Other: "hypertension care trajectories that are not compatible with the cascade of care framework (no awareness, but treatment, control or not)". We also specified that each individual could only be in one of these steps of hypertension management. We changed the figures, tables, and result sections accordingly (and added notes to tables and figures to clarify the definitions).**

**Our analytical approach was intentionally designed to model the progression through the care cascade using a multinomial regression framework, which allows us to simultaneously assess the relative likelihoods of different steps of hypertension management in a unified model, while also accommodating the interdependence among them. While presenting results for awareness, treatment, and control as separate outcomes would certainly be more straightforward in terms of readability and interpretation, to understand, they can introduce selection bias and reduce statistical power, especially when subgroups are small (as is our case). Our multinomial model avoids these issues by analyzing all outcomes relative to a common reference among all those identified with hypertension. However, as suggested by the other reviewer, we added marginal probabilities tables that help clarify the results.**

**Thank you for underlining the lack of definition of the "other" step. We added a definition in the methods section: "In the cascade of care framework, we determined that individuals were in "Other" step, if they had reached treatment (and control or not), without reaching awareness."**

Abstract:
- Include the number of women in the cohort and provide percentages for each ethnic group.

**Added information in the participants section of the abstract:**

**"180,459 individuals were included, aged 18-69 (mean age 47 years), among which 53% (n=95,395) women and 81% (n=145,983) of the majority group, and 4.9% (n=8,775) of North Africans, 1.2% (n=2,220) of Subsaharan African, 1.2% (n=2,204) of Asian, 1.4% (n=4,462) of Overseas France *départements* and regions (DROMs), and 10% of European and other descents. Among these 180,459 individuals,54,009 (29.9%) had hypertension."**

- Add the mean age of participants.

**Added information in the participants section of the abstract:**

**"180,459 individuals were included, aged 18-69 (mean age 47 years), among which 53% (n=95,395) women and 81% (n=145,983) of the majority group, and 4.9% (n=8,775) of North Africans, 1.2% (n=2,220) of Subsaharan African, 1.2% (n=2,204) of Asian, 1.4% (n=4,462) of Overseas France *départements* and regions (DROMs), and 10% of European and other descents. Among these 180,459 individuals,54,009 (29.9%) had hypertension."**

Introduction:
The introduction is currently too long and could benefit from a more concise, focused presentation.

**We deleted some sentences in the introduction, and edited the text to tighten-up the language:**

**"~~Despite some recent progress data on hypertension epidemiology and management in European immigrant patients are still lacking"~~**

**~~Recently, the European Society of Cardiology (ESC) calls for action for similar studies on ethnoracial differences in the prevalence and management of hypertension in European countries[3]~~**

**~~even after considering socioeconomic status[9], and argue that similar studies should be conducted in other European countries~~**

Methods:
- The definition of ethnicity is unclear. While migration status is used as a proxy, it is important to clarify how individuals of mixed ethnic background were categorized and how it is based on the migration status of their parents.

**Supplementary Figure 1 (Migration status categorization in CONSTANCES) was added in the supplementary material. Furthermore, a note under the figure was also added to provide additional details on individuals with mixed ethnic background: For descendants of immigrants (or natives of DROMs), if they are**

**born to a mixed-origin couple, consisting of two migrant parents of different origins, they are assigned the father's origin, for the sake of simplicity.**

**We also added theorical arguments to how we coded the variable, and how it can be considered as a proxy for race/ethnicity in the methods section.**

- Please clarify what is meant by "overseas France," as this term encompasses a range of regions and populations.

**We added a sentence in the methods section: "Overseas France consists of French territories located outside Europe, largely made up of the remaining parts of the former French colonial empire that continued to be integrated into the French state under different legal statuses after decolonization".**

**A footnote was added to that sentence: "Among these territories, the most populated ones (over 200,000 inhabitants in 2021) are Guadeloupe, Martinique, French Guiana, Réunion, Mayotte, French Polynesia, and New Caledonia."**

- How were discrepancies between self-reported data and registry data handled? For instance, history of cardiovascular disease was assessed through both self-report and the SNDS.

**We added a sentence to explain how self-report data was used to complete registry data that was only available for the 2007-2021 period.**

**"History of other CVD (namely coronary disease, myocardial infarction, stroke, transient ischemic attack, peripheral artery disease, heart failure) was either reported when answering the doctor-administered questionnaire or identified in the SNDS. Using self-reported data in CONSTANCES enabled us to complete the registry data (SNDS) that only recorded medical reimbursements or hospitalizations for one of the aforementioned CVD between 2007 and 2021".**

- Please add a participant flowchart (as supplementary material) to clarify the selection process.

**A flowchart has been provided in the Supplementary Material (Supp Fig 1)**

Results:

- Please begin the Results section with descriptive statistics: mean age, number of women, and the ethnic distribution of the cohort.

**We added the descriptive statistics at the beginning of the Results section:**

**"Baseline characteristics of the CONSTANCES cohort by race/ethnicity and sex are shown Table 1. Briefly, women made up 53% of the cohort, and 19% of participants belonged to an ethnoracial minority (4.9% of North African, 1.2% of Subsaharan African, 1.2% of Asian, 1.4% of DROMs, and 10% of European and Other descents), with no significant differences between women and men. The mean age was 47 years old (standard deviation (SD): 13)."**

**We also added similar information on the CONSTANCES population identified as having hypertension:**

**"Among the 180,459 individuals included in our study, 29.9% (n=54,009) had hypertension (mean age 55 (SD:11), 39% women and 3.6% (n=1,933) of North African, 1.4% (n=758) of SSA, 0.8% (n=429) of Asian, 1.3% (n=697) of DROMs, 10% of European and Other (n=5,564) descents, and 83% (n=44,628) of the majority group))."**

- The "other" step is not clearly defined and lacks clinical relevance. It may be better to omit this and present the outcomes (awareness, treatment, control) separately.

**In the cascade of care framework, we determined that individuals were in "Other" step, if they had reached treatment (and control or not), without reaching awareness. We added a definition in the methods section.**

**We agree that this "other" category may be challenging to interpret clinically. However, we have chosen to retain this group for several reasons:**

- **Inclusivity of the full population identified as having hypertension: our aim is to examine hypertension management path among all individuals identified with hypertension, regardless of their awareness status. Excluding this group will omit a segment of the treated hypertensive population that did not self-report hypertension.**
- **As noted in prior research (Silberzan, 2024), individuals who are treated but unaware often represent a socially patterned subgroup. We wanted to avoid selection biases.**
- **While more difficult to categorize clinically, this group is meaningful from a population health perspective by acknowledging the limits of the cascade of care framework (such as those discussed in the discussion section).**

**Our response on presenting outcomes separately can be found above in the overall comments.**

Tables and Figures:
- Table 1: Add the total number of participants in each category.

**We added the total number of participants in each category**

- For Figures 3 and 4, add brief explanations of the steps underneath the figures for reader clarity.

**Explanations were added underneath the figures**

- As noted earlier, consider removing the "other" step, especially since Figure 1 notes that it is not compatible with clinical interpretation.

**Response to this comment can be found in the 2nd comment of the result section.**

Discussion:
- The discussion focuses on comparisons with studies from the USA. Please consider referencing to European studies (the HELIUS study for example), as population characteristics of European countries are more comparable.

**As suggested, we added references to European studies, namely from the Netherlands and the UK.**

**To be more precise, we modified the in the introduction:**

**While ethnoracial disparities in hypertension prevalence are documented in other high-income countries, <u>such as the United States [7], the Netherlands, or the UK [22,23],</u> it is not the case for France.**

**And added the following references:**

**[7] Aggarwal R, Chiu N, Wadhera RK, et al. Racial/Ethnic Disparities in Hypertension Prevalence, Awareness, Treatment, and Control in the United States, 2013 to 2018. Hypertension. 2021;78:1719–26. doi: 10.1161/HYPERTENSIONAHA.121.17570**
**[22] Modesti PA, Reboldi G, Cappuccio FP, et al. Panethnic Differences in Blood Pressure in Europe: A Systematic Review and Meta-Analysis. PLoS One. 2016;11:e0147601. doi: 10.1371/journal.pone.0147601**

**[23] van Laer SD, Snijder MB, Agyemang C, et al. Ethnic differences in hypertension prevalence and contributing determinants - the HELIUS study. Eur J Prev Cardiol. 2018;25:1914–22. doi: 10.1177/2047487318803241**

**We also verified that we cited European studies in the rest of the discussion, such as the following:**

**"Our findings are consistent with results on cohorts from the UK and the Netherlands[8] suggesting, among other factors, that populations from African nations might also experience inequalities in access to healthcare compared to the majority group, ultimately impacting control as well."**

**[8] van der Linden EL, Couwenhoven BN, Beune EJAJ, et al. Hypertension awareness, treatment and control among ethnic minority populations in Europe: a systematic review and meta-analysis. J Hypertens. 2021;39:202–13. doi: 10.1097/HJH.0000000000002651**

- Similar to the introduction, the discussion is too long, please discuss your results more to-the-point.

**We shortened the discussion section and edited the text:**

~~**In fact, control via the "no awareness, treatment, control" path has proven to be associated with having pre-existing CVD in the CONSTANCES cohort[34].**~~

~~**A recent study using the intersectionality framework - a framework that considers the simultaneity of different social dimensions, such as age, gender and ethnicity- in the US[26] shows that Black women fare worse than Black men while White women have lower risk of hypertension than White men. Furthermore, at age 51, faced with multiple health disadvantages from early life onwards, Black women have the highest hypertension prevalence rates compared to other race/ethnicity-gender groups**~~

~~**The 2023 ESH (European Society for Hypertension) Guidelines and the 2024 ESC Guidelines for the management of elevated blood pressure and hypertension**~~

~~**a study on a French nationally representative survey in 2020 shows that**~~

- Provide an explanation why such a small proportion of individuals are treated, aware, or in control of their hypertension (only 12.9%).

**We added a paragraph before the limitations section to provide possible explanations to why the control rate was low in France, compared to other European countries.**

**“Overall, our study found that only 12.9% are aware, treated, and controlled, which is low compared to other high-income countries[5]. This can be attributed to several interconnected factors. First, the treatment rate has been declining in recent years among women [49]. This trend is compounded by a notable changes in therapeutic approaches: the proportion of patients on monotherapy increased from 45% in 2014 to 55% in 2019 , implying a corresponding reduction in the use of combination therapies, which are often more effective for blood pressure control [50]. Not to mention that fixed-dose triple combinations -i.e, three antihypertensive drugs combined in a single pill- shown to be associated with higher control rates than three separate pills [51,52] were not reimbursed in France before 2025, an exception among European countries [53]. Furthermore, policy changes such as the removal of full health coverage for severe hypertension under the "Affection de Longue Durée" (ALD) status—formerly allowing 100% reimbursement for chronic conditions—have reduced access to free care to individuals diagnosed after 2011, possibly impacting control [53]. Reimbursement is also lacking for home blood pressure monitoring devices, creating financial barriers for optimal management[54]. Finally, according to a survey on a sample of 753 GPs in France, less than 50% of them reported feeling highly implicated in dietary prevention or overweight and obesity [55], often citing lack of time and resources, which further hinders effective blood pressure control [56].”**

**Added references:**

**[49]Perrine, Anne-Laure. « L’hypertension artérielle en France: prévalence, traitement et contrôle en 2015 et évolutions depuis 2006 ». Bulletin Epidémiologique Hebdomadaire, no 10 (2018).**

**[50]Wald, David S., Malcolm Law, Joan K. Morris, Jonathan P. Bestwick, et Nicholas J. Wald. « Combination Therapy Versus Monotherapy in Reducing Blood Pressure: Meta-Analysis on 11,000 Participants from 42 Trials ». The American Journal of Medicine 122, no 3 (mars 2009): 290-300. https://doi.org/10.1016/j.amjmed.2008.09.038.**

**[51]Koval, Sergiy M., Iryna O. Snihurska, Tetyana G. Starchenko, Marina Yu. Penkova, Olga V. Mysnychenko, Kostyantin O. Yushko, Olga M. Lytvynova, Olena Vysotska, et Alexander E. Berezin. « Efficacy of Fixed Dose of Triple Combination of Perindopril-Indapamide-Amlodipine in Obese Patients with Moderate-to-Severe Arterial Hypertension: An Open-Label 6-Month Study ». Biomedical Research and**

**Therapy 6, no 11 (30 novembre 2019): 3501-12. https://doi.org/10.15419/bmrat.v6i11.578.**

**[52]Nedogoda, Sergey V., et Vesna J. Stojanov. « Single-Pill Combination of Perindopril/Indapamide/Amlodipine in Patients with Uncontrolled Hypertension: A Randomized Controlled Trial ». Cardiology and Therapy 6, no 1 (1 juin 2017): 91-104. https://doi.org/10.1007/s40119-017-0085-7.**

**[53]Rubin, Sébastien, Romain Boulestreau, Thierry Couffinhal, Christian Combe, et Xavier Girerd. « La dégradation inquiétante du contrôle de l'hypertension artérielle en France : notre alerte à destination des néphrologues ». Néphrologie & Thérapeutique 16, no 6 (novembre 2020): 347-52. https://doi.org/10.1016/j.nephro.2020.10.001.**

**[54]Cappuccio, Francesco P, Sally M Kerry, Lindsay Forbes, et Anna Donald. « Blood pressure control by home monitoring: meta-analysis of randomised trials ». BMJ : British Medical Journal 329, no 7458 (17 juillet 2004): 145. https://doi.org/10.1136/bmj.38121.684410.AE.**

**[55]Grave, Clémence. « Prevention, screening and management of hypertension in France, the point of view of general practitioners, France, 2019 ». Bull Epidémiol Hebd 2020, no 5 (2020): 115-23.**

**[56]Julius, Stevo, Mariaconsuelo Valentini, et Paolo Palatini. « Overweight and Hypertension ». Hypertension 35, no 3 (mars 2000): 807-13. https://doi.org/10.1161/01.HYP.35.3.807.**

Reviewer: 2
Dr. Lirui Jiao, The University of North Carolina at Chapel Hill Gillings School of Global Public Health
Comments to the Author:
This study investigates the intersection of sex and ethnicity in hypertension prevalence and care outcomes in France, using data from over 180,000 adults in the CONSTANCES cohort linked to national health records. By using migration status as a proxy for ethnicity, the authors examined age-standardized hypertension prevalence and progression through the care cascade (i.e., awareness, treatment, and control) via multinomial logistic regression. The findings reveal significant disparities, where individuals from Sub-Saharan Africa and Overseas France, particularly women, have higher hypertension prevalence and are more likely to remain in treatment without achieving control. Other groups, such as women of European/other origin and Asian men, are more likely to remain in the aware step. The study highlights the importance of tailoring hypertension interventions to both ethnic and sex-specific patterns.

**We would like to thank Dr Lirui Jiao for the time and effort put in reviewing this article. Please find below the responses to the comments.**

Introduction

The introduction of the study provides a strong rationale for the research by clearly establishing hypertension as a significant public health concern globally. It effectively contextualizes the importance of examining disparities across the care cascade and frames ethnicity, examined using migration status as a proxy, as a key yet underexplored determinant in France. The authors provide a comprehensive overview of literature from the U.S. and other European countries and emphasize the lack of comparable data in France due to legal constraints on collecting ethnic data.

1. The last sentence in the third paragraph (Line 55-56) contains errors and need to be revised for clarity.

**The sentence has been changed to: "While studies are still scarce, a recent systematic review[8], mainly using data from cohorts in the UK and the Netherlands (none from France), finds race/ethnicity inequalities among men and women in the hypertension management cascade, even after considering socioeconomic status[9], and argue that similar studies ~~are~~ should be conducted in other European countries ~~and should be conducted~~."**

2. The authors could state more explicitly the contribution of the present study relative to prior French studies, beyond simply addressing a gap in the literature.

**We added sentences in the introduction to underline the contribution of our study:**

**While those studies are still scarce, a systematic review[8], mainly using data from cohorts in the UK and the Netherlands (none from France), finds race/ethnicity inequalities among men and women, although age could not be taken into account.**

3. In addition, while the authors note that only two clinical studies have examined hypertension by migration status, it is unclear how this study expands upon those studies.

**We rephrased the end of the paragraph mentioning the two clinical studies, so as to underline how our study goes further. We also added some elements in the main objective:**

**"However, these results cannot be generalized to the general population, the studies are limited to North Africans and SSA and do not investigate other**

**migratory groups (such as DROMs or Asians), and being referred to a specialist hypertension unit may be socially determined and associated with greater hypertension awareness."**

**To address existing gaps in the evidence from France, our study describes the prevalence of hypertension and each stage of the hypertension care cascade within the general population, at the intersection of sex, age, and a wide range of ethnoracial groups."**

4. The authors do a good job of explaining why ethnic data is difficult to obtain, however, they should provide further context on the current proportions of the ethnicity groups among the French population and explicate more clearly why migration status is a reasonable proxy.

**As suggested, we added some elements in the introduction:**

**"However, in a country where immigrants and natives from DROMs make up around 14% (among which around 8% from DROMs, 30% from North Africa, 13% from Subsaharan Africa, 19% from Europe, and 11% from Asia) of the Metropolitan French population and descendants of immigrants and of natives from DROMs around 13% (among which around 10% from DROMs, 6% Subsaharan Africa, 36% from Europe, and 7% from Asia), [13], migration status is a different variable that may act as an imperfect proxy for race/ethnicity. Migration status is strongly correlated with race/ethnicity and more commonly collected and available in routine data in France and can help identify populations that may be disproportionately exposed to discrimination related to racism and xenophobia[14]"**

Methods

The use of the CONSTANCES cohort enhances generalizability to the French adult population aged 18–69. The care cascade logic (control implies treatment, treatment implies awareness) is clearly articulated and consistent with prior literature. Appropriate statistical approaches were also used. However, there are a few weaknesses that require attention.

1. The authors should discuss the limitations of using migration status as an imprecise proxy for ethnicity in this section. It would also be helpful to cite prior studies that have used this approach if possible.

**In the discussion section, we cited prior studies using this approach:**

**Second, even though migratory status has proven to be a valid proxy for race/ethnicity[13] <u>and has been used in French health research [58,59]</u>, it is important to emphasize that the two are not equivalent**

**We also discussed the limits of using this approach in the discussion section:**

**"Furthermore, this approach limits our ability to capture the experiences of third- and fourth-generation individuals, who may no longer be classified as belonging to an ethnoracial minority but continue to face racial discrimination and its associated health impacts[13,14]. Relying on migratory status also blurs the line between the social experience of migration and that of racialization and racism. While both can intersect, they represent distinct processes with different social and health consequences [14]."**

**Added references:**

**[13]Simon, Patrick. « Statistics, French Social Sciences and Ethnic and Racial Social Relations »: Revue Française de Sociologie Vol. 51, no 5 (15 janvier 2011): 159-74. https://doi.org/10.3917/rfs.515.0159.**

**[14] Brun, Solène, et Claire Cosquer. « Sociologie de la race », 1 septembre 2022. https://doi.org/10.3917/arco.brun.2022.01.**

**[58]Bajos, Nathalie, Emilie Counil, Jeanna-eve Franck, Florence Jusot, Ariane Pailhé, Alexis Spire, Claude Martin, et al. « Social Inequalities and Dynamics of the Early COVID-19 Epidemic: A Prospective Cohort Study in France ». BMJ Open 11, no 11 (1 novembre 2021): e052888. https://doi.org/10.1136/bmjopen-2021-052888.**

**[59]Bajos, Nathalie, Alexis Spire, Léna Silberzan, Antoine Sireyjol, Florence Jusot, Laurence Meyer, Jeanna-Eve Franck, et al. « When Lack of Trust in the Government and in Scientists Reinforces Social Inequalities in Vaccination Against COVID-19 ». Frontiers in Public Health 10 (2022). https://www.frontiersin.org/articles/10.3389/fpubh.2022.908152.**

2. Although education is included, the authors should consider also controlling for other socioeconomic variables, such as income, employment, etc, to address confoundedness.

**We acknowledge the reviewer's comment regarding checking for potential confounders. We evaluated several candidate variables for potential confounding, (such as education, employment status, reported financial hardship), but none met the necessary criteria of being associated with both the exposure and the outcome without being on the causal pathway. As such, no confounders were identified.**

**The primary objective of our study was to provide a descriptive overview of the observed differences, rather than to investigate the underlying mechanisms. While we agree that further adjustment could offer additional insights, such analyses fall outside the intended scope of this work.**

**We added a sentence in the methods section: "As the objective of our study was to provide a descriptive overview of the distribution prevalence of hypertension and each stage of the hypertension care cascade at the intersection of sex, age, and race/ethnicity, we did not investigate causal mechanisms. Therefore, we did not adjust for other variables than age and race/ethnicity, to avoid overadjustment bias [21]."**

**Added reference:**

**[19] Zwieten, Anita van, Peter W. G. Tennant, Michelle Kelly-Irving, Fiona M. Blyth, Armando Teixeira-Pinto, et Saman Khalatbari-Soltani. « Avoiding overadjustment bias in social epidemiology through appropriate covariate selection: a primer ». Journal of Clinical Epidemiology 149 (1 septembre 2022): 127-36. https://doi.org/10.1016/j.jclinepi.2022.05.021.**

3. The authors could consider adding a flow chart to show how they obtained their final sample.

**A flowchart has been provided in the Supplementary Material (Supp Fig 1)**

Results
The results section presents a comprehensive and well-structured summary of findings, with clear reporting of descriptive statistics and regression estimates, stratified by sex and ethnic groups. However, some areas could be improved to strengthen interpretability and rigor.

1. While reporting odds ratios is appropriate for multinomial logistic regression, the use of odds ratio limits interpretability for the audience. The authors could improve upon this by acknowledging the limitations of interpreting ORs as risks, especially when outcomes are common, and by providing additional estimates (e.g., marginal probabilities or absolute differences) to enhance interpretability and policy relevance.

**Thank you for this comment, as we believe it has helped the article gain clarity and statistical rigor. As suggested, we added a paragraph to explain the limits of interpreting ORs as risks, and we provided marginal probabilities:**

**"Furthermore, while odds ratios (ORs) are a valid measure of association in logistic regression, they become increasingly difficult to interpret as intuitive measures of risk when the outcome is common (as can be the case in our analyses). In such cases, ORs can substantially overstate the strength of association compared to differences in predicted probabilities, potentially leading to misleading conclusions if interpreted as direct measures of risk. That is why we added marginal predicted**

**probabilities (adjusted for age) to provide a clear and interpretable summary of ethnoracial differences (by sex) in the hypertension care cascade."**

**The marginal probabilities can be found in the Supplementary Material (Supp Tables 7a and b)**

**The result section was also changed, adding age-adjusted marginal predicted probabilities to complete ORs.**

2. The authors should clarify whether education, overweight/obesity, or other covariates were included in sensitivity models. The results do not report whether odds ratios differ significantly across subgroups beyond sex (e.g., interaction terms by migration group and education level).

**The decision to conduct a stratified analysis by sex was based on theoretical considerations aimed at elucidating potential sex-specific differences, rather than being driven by a statistical interaction term. As the objective of the article was to describe prevalence and hypertension cascade of care position at the intersection of age, sex, and race/ethnicity, we did not add other covariates in analyses.**

**We added a sentence in the methods section: "As the objective of our study was to provide a descriptive overview of the distribution prevalence of hypertension and each stage of the hypertension care cascade at the intersection of sex, age, and race/ethnicity, we did not investigate causal mechanisms. Therefore, we did not adjust for other variables than age and race/ethnicity, to avoid overadjustment bias [21]."**

**Added reference:**

**[19] Zwieten, Anita van, Peter W. G. Tennant, Michelle Kelly-Irving, Fiona M. Blyth, Armando Teixeira-Pinto, et Saman Khalatbari-Soltani. « Avoiding overadjustment bias in social epidemiology through appropriate covariate selection: a primer ». Journal of Clinical Epidemiology 149 (1 septembre 2022): 127-36. https://doi.org/10.1016/j.jclinepi.2022.05.021.**

Discussion

The discussion section offers a thoughtful and well-contextualized interpretation of the study's findings. However, the authors should revise their current sentence "These studies show that individuals of African origin in Europe (resp. African Americans in the US) consistently have higher hypertension rates, putting forward biological hypotheses, such as early arterial stiffening, higher salt-sensitivity and propension to overweight and obesity" to avoid implying a biological determinism linked to ethnicity.

**We changed the sentence and added more precise elements from the literature, to avoid implying a biological determinism linked to race/ethnicity:**

**"Whilst there may be genetic markers of predisposition to hypertension in African populations -although intensive investigations into genetic links have yielded less conclusive results than anticipated [2]- they are not sufficient on their own to cause hypertension or elevate risk [24]. Social factors play a critical role in this higher burden [22,24].~~On the other hand, the higher burden of hypertension among African populations are highly modifiable with diet and lifestyle changes [22,24], and social factors also play a critical role in this higher burden [22,24]. [2][2][22,24].. More precisely~~ Specifically, structural racism [25], ~~putting forward biological hypotheses, such as early arterial stiffening, higher salt-sensitivity and propension to overweight and obesity[2,3]. Social factors, namely~~ socioeconomic disadvantage, discrimination, differential treatments in the healthcare system, and chronic stress[26] increase hypertension risk -such as overweight and obesity- [27] and ~~are also suggested~~ underlie the increased risk of high BP from younger ages in these populations.**

**[…]**

**Furthermore, in the CONSTANCES cohort, the high overweight/obesity rates and the over-representation in the worst DASH tercile (Table 1) -also influenced by social factors such as structural racism [33,34]- could contribute to explaining the higher BP rates of women of SSA and DROMs ~~Overseas France~~, compared to the majority group."**

**Added references:**

**[22]Modesti, Pietro Amedeo, Gianpaolo Reboldi, Francesco P. Cappuccio, Charles Agyemang, Giuseppe Remuzzi, Stefano Rapi, Eleonora Perruolo, Gianfranco Parati, et ESH Working Group on CV Risk in Low Resource Settings. « Panethnic Differences in Blood Pressure in Europe: A Systematic Review and Meta-Analysis ». PLOS ONE 11, no 1 (25 janvier 2016): e0147601. https://doi.org/10.1371/journal.pone.0147601.**

**[24]Jobe, Modou, Serigne Mor Beye, Ngone Diaba Gaye, Mame Madjiguene Ka, Pablo Perel, Alexander D. Perkins, Adama Kane, et al. « Hypertension in Sub-Saharan Africa: Burden, Barriers and Priorities for Improving Treatment Outcomes ». Circulation Research 137, no 1 (20 juin 2025): 106-18. https://doi.org/10.1161/CIRCRESAHA.124.323889.**

**[25]Bailey, Zinzi D, Nancy Krieger, Madina Agénor, Jasmine Graves, Natalia Linos, et Mary T Bassett. « Structural racism and health inequities in the USA: evidence**

**and interventions ». The Lancet 389, no 10077 (8 avril 2017): 1453-63. https://doi.org/10.1016/S0140-6736(17)30569-X.**

**[26]Richardson, Liana J., et Tyson H. Brown. « (En)Gendering Racial Disparities in Health Trajectories: A Life Course and Intersectional Analysis ». SSM - Population Health 2 (1 décembre 2016): 425-35. https://doi.org/10.1016/j.ssmph.2016.04.011.**

**[27]Brondolo, Elizabeth, Erica E. Love, Melissa Pencille, Antoinette Schoenthaler, et Gbenga Ogedegbe. « Racism and Hypertension: A Review of the Empirical Evidence and Implications for Clinical Practice ». American Journal of Hypertension 24, no 5 (1 mai 2011): 518-29. https://doi.org/10.1038/ajh.2011.9.**

**[33]Bell, Caryn N., Jordan Kerr, et Jessica L. Young. « Associations between Obesity, Obesogenic Environments, and Structural Racism Vary by County-Level Racial Composition ». International Journal of Environmental Research and Public Health 16, no 5 (9 mars 2019): 861. https://doi.org/10.3390/ijerph16050861.**

**[34]Dougherty, Geoff B., Sherita H. Golden, Alden L. Gross, Elizabeth Colantuoni, et Lorraine T. Dean. « Measuring Structural Racism and Its Association With BMI ». American Journal of Preventive Medicine 59, no 4 (1 octobre 2020): 530-37. https://doi.org/10.1016/j.amepre.2020.05.019.**

Overall, this study provides a large-scale and timely examination of the hypertension care cascade by sex and ethnicity, using a large, population-based French cohort. However, revisions are needed to improve clarity and interpretability in the method and results sections, and authors should take caution to not conflate ethnicity with biological constructs in the discussion section. Furthermore, authors should check for grammatical errors throughout the manuscript.

**We would like to underline that we also changed “ethnic” to “ethnoracial” and “ethnicity” to “race/ethnicity”, as we believe that the latter term better captures Social and Structural Realities, avoids oversimplification, and better reflects how racial and ethnic dimensions can intersect.**

**We hope that our revisions and explanations address your concerns and that the manuscript is now suitable for publication. Thank you again for your valuable feedback and the opportunity to revise our work.**

---

## VERSION 2 - REVIEW

---

| | |
|---|---|
| **Reviewer** | **1** |
| **Name** | **Vriend, Esther** |
| **Affiliation** | **University of Amsterdam** |

**Date** **09-Aug-2025**

**COI**

---

The authors have thoroughly addressed all the feedback provided. I find the revisions to the methodology clear and well-justified. Additionally, the improvements made to (especially) the abstract and methods section enhance the clarity and focus of the paper. Overall, I am satisfied with the changes and believe the manuscript has significantly improved as a result.
